# Field Experiences with Handheld Diagnostic Devices to Triage Children under Five Presenting with Severe Febrile Illness in a District Hospital in DR Congo

**DOI:** 10.3390/diagnostics12030746

**Published:** 2022-03-18

**Authors:** Bieke Tack, Daniel Vita, Irène Mansosa, Thomas Nsema Mbaki, Naomie Wasolua, Aimée Luyindula, Jaan Toelen, Octavie Lunguya, Jan Jacobs

**Affiliations:** 1 Department of Clinical Sciences, Institute of Tropical Medicine, 2000 Antwerp, Belgium; jjacobs@itg.be; 2 Department of Microbiology, Immunology and Transplantation, KU Leuven, 3000 Leuven, Belgium; 3 Hôpital Général de Référence Saint Luc de Kisantu, Kisantu, Democratic Republic of the Congo; dvitamayimona@gmail.com (D.V.); irenemansosa2021@gmail.com (I.M.); thomasnsema202020@gmail.com (T.N.M.); naomiewasolua@gmail.com (N.W.); aimeeluyindula1@gmail.com (A.L.); 4 Department of Development and Regeneration, KU Leuven, 3000 Leuven, Belgium; jaan.toelen@kuleuven.be; 5 Department of Microbiology, Institut National de Recherche Biomédicale, Kinshasa, Democratic Republic of the Congo; octmetila@yahoo.fr; 6 Department of Medical Biology, University Teaching Hospital of Kinshasa, Kinshasa, Democratic Republic of the Congo

**Keywords:** handheld diagnostic device, triage, low-resource setting, severe febrile illness, danger sign, usability, label comprehension, instructions for use, children under five

## Abstract

As part of a field study (NCT04473768) in children presenting with severe febrile illness to Kisantu hospital (DR Congo), we retrospectively compiled user experiences (not performance) with handheld diagnostic devices assisting triage: tympanic thermometer, pulse oximeter (measuring heart rate, respiratory rate and oxygen saturation), hemoglobinometer and glucometer. Guidance documents for product selection were generic and scattered. Stock rupture, market withdrawal and unaffordable prices interfered with procurement. Challenges at implementation included environmental temperature, capillary blood sampling (antisepsis, order of multiple tests, filling microcuvettes and glucose strips), calibration (environmental temperature, cold chain) and liability-oriented communication with a manufacturer. Instructions for use were readable and contained symbol keys; two devices had printed French-language instructions. Shortcomings were poor integration of figures with text and distinct procedures for the oximeter and its sensor. Usability interview revealed appreciations for quick results, visibility of the display and memory function (three devices) but also problems of capillary blood sample transfer, cleaning, too long of a time-to-results (respiratory rate) and size, fitting and disposal of thermometer probes. Pictorial error messages were preferred over alphanumeric error codes but interpretation of symbols was poor. Alarm sounds of the oximeter caused unrest in children and caretakers perceived the device as associated with poor prognosis.

## 1. Introduction

Severe febrile illness is a major global cause of under-five mortality and affects particularly children in sub-Saharan Africa [1,2,3]. Severe febrile illness includes malaria and bloodstream infection, which require prompt referral to hospital [2,4] where triage is done to identify those in need for prompt antimalarial, antibiotic and supportive treatment [5,6]. Decisions at referral and triage are mainly based on the presence of clinical danger signs such as temperature, heart rate, respiratory rate, oxygen saturation, blood glucose and hemoglobin levels [5,6]. However, frontline healthcare workers frequently overlook these danger signs due to limited skills and experience and non-availability of diagnostics. Missing danger signs implies delays or non-referral at the healthcare level and late or inappropriate treatment at the hospital level, increasing mortality and morbidity [2].

**Figure 1:**
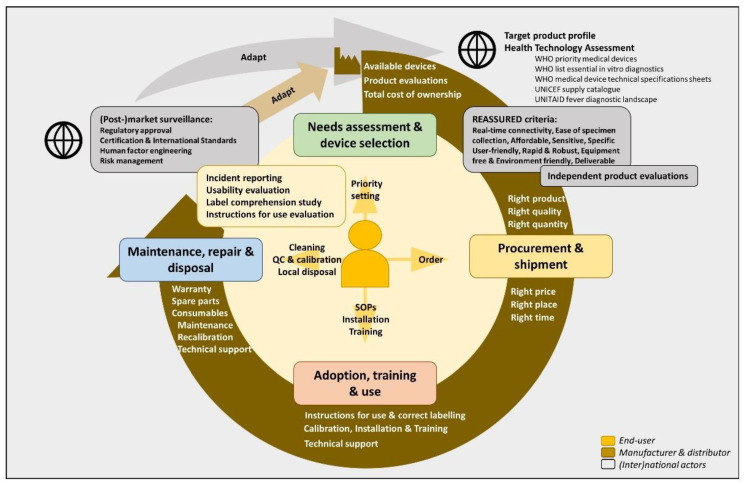
Life cycle of a diagnostic device at the end-user’s level as described by WHO. At the end-user level, it starts with selection of the diagnostic device based on a needs assessment. Target product profiles, technical specification sheets and independent product evaluations based on the REASSURED criteria provide guidance for selection. Close interaction with the manufacturer and a healthy market is pivotal for the phase of procurement and shipment. Subsequent adoption, training and use rely on user-adapted instructions for use while manufacturer’s support needs to continue during the maintenance, repair and disposal. End-user’s experiences should be integrated in post-market surveillance and feed improvements in device and service. For an extended version of this figure, we refer to Supplement S1. Abbreviations: SOP: Standard Operating Procedure; QC: quality control.

To facilitate recognition of danger signs, the World Health Organization (WHO) recommends the use of handheld diagnostic devices. These devices are used bedside and provide results within a few minutes. Examples are an oximeter to detect hypoxia or a glucometer to detect hypoglycemia. To improve rolling-out of diagnostic technologies, WHO developed recommendations that cover the complete life cycle of the handheld diagnostic devices, from development to disposal (Figure 1 and Supplement S1) [7].

In this retrospective study, we compiled obstacles experienced throughout the lifecycle of handheld diagnostic devices used for hospital triage of children under five-years-old presenting with severe febrile illness in a low-resource setting. Experiences were reported from an end-user’s perspective with special attention for available guidance for selection of medical devices, instructions for use, usability and label comprehension.

## 2. Materials and Methods

### 2.1. Study Setting, Design and Period

**Figure 2:**
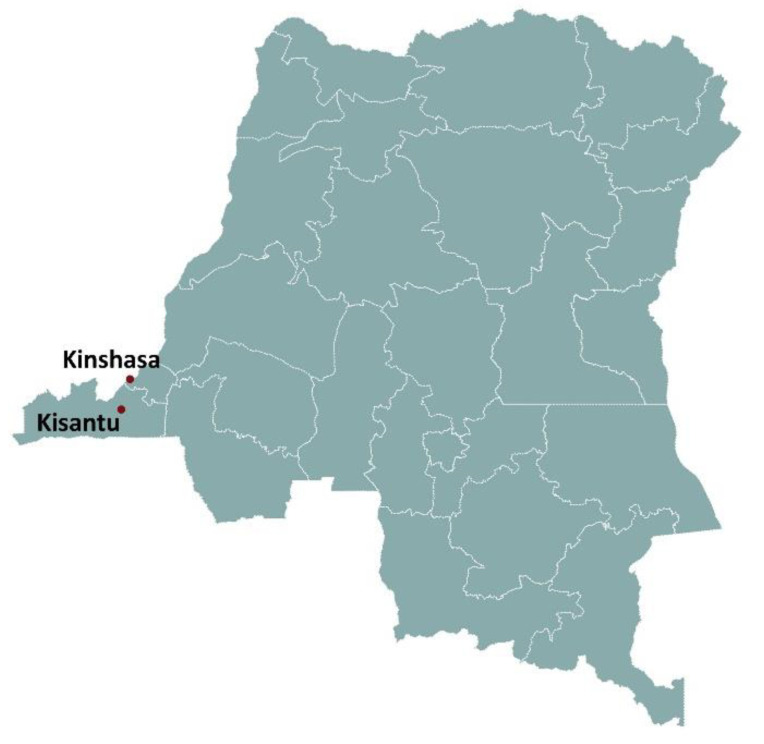
Geographical location of Kisantu hospital in DR Congo.

Reference Hospital St. Luc in Kisantu (further named Kisantu Hospital) is a district referral hospital located in Kongo Central Province in the Democratic Republic of Congo (DR Congo, Figure 2). The health care setting in DR Congo is characterized by its poor infrastructure and health services [8]. Kisantu has a tropical climate with average monthly temperatures up to 27 °C and an average monthly humidity up to 80% [9,10,11]. The local burden of *Plasmodium falciparum* malaria, invasive *Salmonella* infections, anemia and malnutrition is high in children under five-years-old [8,9,12,13]. Kisantu hospital has about 340 beds with high bed occupancy rates in the pediatric ward [14]. It is part of a national microbiological surveillance network [8,12]. Blood cultures are integrated in the routine clinical practice and sampled and worked up free of charge [8,12]. The presence of a flat hospital rate [14] and the affordability of the microbiology laboratory allows Kisantu hospital to host clinical studies [8,12].

The present study fits into a prospective study on the clinical presentation of children admitted to hospital with invasive *Salmonella* infections (DeNTS study: NCT04473768). Children recruited were >28 days and <5-years-old. Triage occurred during eligibility screening (temperature measurement) and first examination (heart rate, respiratory rate, oxygen saturation, hemoglobin and blood glucose measurement) by dedicated research nurses. Educational level, auxiliary and language skills of research nurses were representative for those of local healthcare workers.

As part of triage in the period before the study, temperature was measured with a digital axillary thermometer, heart rate and respiratory rates were counted manually and oxygen saturation, hemoglobin and blood glucose were not routinely measured. Malaria was diagnosed by microscopy and rapid diagnostic tests and blood cultures were sampled and worked up on site to diagnose bloodstream infections [8,12].

At the start of the DeNTS study, we selected and implemented handheld diagnostic devices to assist triage and presumptive diagnosis and to document disease severity. For the present study, we retrospectively compiled our experiences with these handheld diagnostic devices during the period February–December 2021.

### 2.2. Lifecycle of Handheld Diagnostic Devices and REASSURED Criteria

Based on the WHO technical series on medical devices [7,15,16,17,18,19,20,21,22,23,24,25,26] and the REASSURED criteria (Real-time connectivity, Ease of specimen collection, Affordable, Sensitive, Specific, User-friendly, Rapid and Robust, Equipment free and Environmentally friendly and Deliverable) [27], we developed a framework that identified key elements along the lifecycle of handheld diagnostic devices at the health-facility level (Figure 1 and Supplemental Figure S1). Field experiences with selected handheld diagnostic devices were reported according to this framework. We did not evaluate the field-performance of the devices, apart from a previously published performance assessment of the Masimo Rad G continuous pulse oximeter to automatically detect the respiratory rate [28].

### 2.3. Needs Assessment: Intended Use of the Handheld Diagnostic Devices and Products

The intended use of the handheld diagnostic devices was measurement and detection of danger signs in children <5-years-old. All danger signs were part of the WHO recommended emergency assessment [5]. End-users were qualified nurses (n = 6) and physicians (n = 2) hired by the DeNTS study. They had no previous experience with the devices used nor with similar devices, except previous experience with a glucometer. The setting was the triage room of the pediatric ward of Kisantu Hospital with a study-dedicated office (3 × 2 m) for recruitment and first exam of patients. The study-dedicated office had electricity supply, but no running water supply.

**Figure 3:**
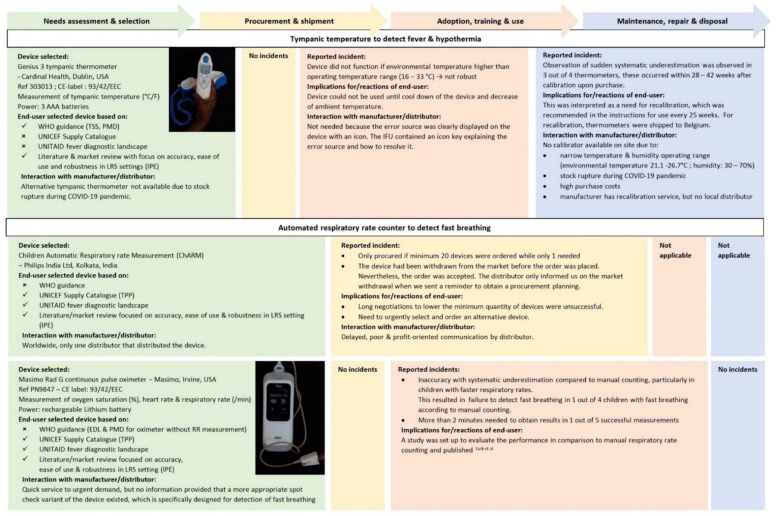
Field experiences reported by end-users during the lifecycle of handheld diagnostic devices to measure tympanic temperature and respiratory rate. Abbreviations and references: IPE: Independent Product Evaluations [38,39,40,41,42,43,44,45,46,47,48,49], PMD: WHO Priority Medical Devices [23], TPP: Target Product Profile [50], TSS: WHO Technical Specification Sheet [24], Tack et al. [28].

**Figure 4:**
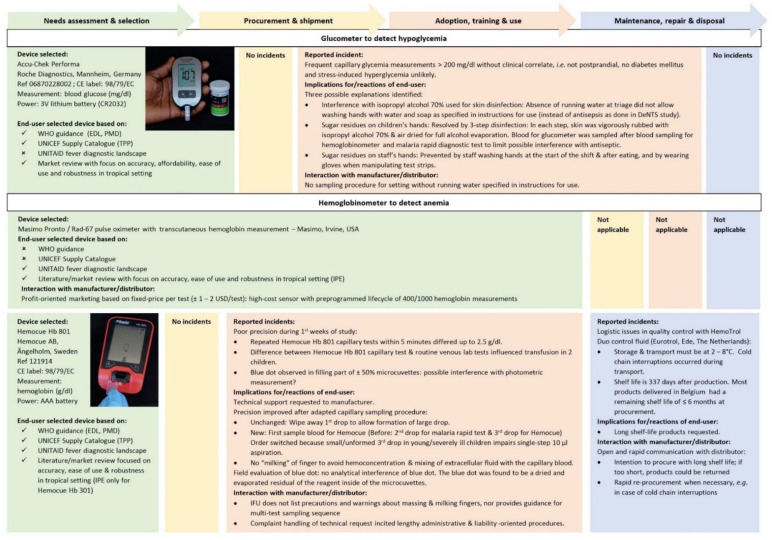
Field experiences reported by end-users during the lifecycle of handheld diagnostic devices to measure blood glucose & hemoglobin. Abbreviations & references: EDL: WHO Essential in vitro Diagnostics List [26], IPE: Independent Product Evaluations [51,52,53,54], TPP: Target Product Profile [55,56,57].

Box 1 lists the medical background and practical considerations for the choice of the danger signs. Figure 3 and Figure 4 list the type, brand and product names and details of the handheld diagnostic devices selected for measuring danger signs as part of the DeNTS study. In addition to the selected devices, we used basic generic devices to complement the clinical exam, i.e., a height board, electronic mother-and-child scale and mid-upper arm circumference (MUAC) tape. These devices were not engineered as small equipment used in triage and, apart from the MUAC tape, not handheld. They were simple and straightforward to use. Therefore, they were not included in the present evaluation. Blood pressure was not performed as it is not recommended by WHO for triage of children: blood pressure measurement in children <3-years-old requires size-adjusted cuffs and age- and height-specific interpretation and has a poor reliability in agitated children [25,29].

Box 1 **Danger signs searched for in children <5-years-old presenting with febrile illness in low-resource settings. The box describes which parameters were used for danger sign detection, the clinical implications of the danger signs and the device by which the parameters were measured. Abbreviations: SOP: Standard Operating Procedure**

**Tympanic temperature—measured by Genius 3 tympanic thermometer (Cardinal Health, Dublin, OH, USA)**
*Danger sign*: fever (>37.5 °C) or hypothermia (≤35.5 °C)
*Procedure*: SOP in Supplement S3. A tympanic thermometer was chosen to avoid the risks of rectal temperature and because of its superior performance compared to other non-invasive thermometers. Cut-offs were set to increase sensitivity and to harmonize with WHO cut-offs recommended for axillary temperature (see Supplement S4).
*Clinical implications*: Fever is a general sign of infection [5]. In infants and children with severe acute malnutrition, severe febrile illness and sepsis can present with hypothermia [5].
**Oxygen saturation—measured by Masimo Rad G continuous pulse oximeter (Masimo, Irvine, CA, USA)**
*Danger sign*: Hypoxia (<90%) [5]
*Procedure:* SOP in Supplement S5
*Clinical implications*: Urgent airway or breathing support needed, e.g. oxygen [5]. Hypoxia is present in severe infections such as pneumonia, sepsis, severe malaria [5,30]. It has a multifactorial pathophysiology including increased oxygen demands, pulmonary inflammation and pulmonary edema [30,31].
**Heart rate—measured by Masimo Rad G continuous pulse oximeter (Masimo, Irvine, CA, USA)**
*Danger sign*: Tachycardia (<12 months: >160/min; 12 months: >120/min) [5]
*Procedure*: SOP in Supplement S5
*Clinical implications*: Often present during fever. If a child has tachycardia, a weak pulse, a cold skin and prolonged capillary refill, the child has poor perfusion/shock requiring fluid resuscitation [5].
**Respiratory rate–measured by Masimo Rad G continuous pulse oximeter (Masimo, Irvine, CA, USA)**
*Danger sign*: fast breathing (<2 months: ≥60 breaths/min; >2−<12 months: ≥50 breaths/min; ≥12 months: ≥40 breaths/min) [5]
*Procedure*: SOP in Supplement S5
*Clinical implications*: Fast breathing can reflect pneumonia, but also be present in severe malaria or invasive bacterial infections due to metabolic acidosis [5].
**Blood glucose–measured by Accu-Chek Performa (Roche Diagnostics, Mannheim, Germany)**
*Danger sign*: hypoglycemia (<45 mg/dL) [5]
*Procedure*: SOP in Supplement S6
*Clinical implications*: Measured on capillary blood to rapidly detect hypoglycemia, as this requires urgent treatment to avert or resolve coma or convulsions [5]. Hypoglycemia (partially) reflects the host stress response to infection with altered activity of the hypothalamic–pituitary–adrenal axis [32,33,34]. As such, hypoglycemia is a diagnostic clue pointing to life-threatening severe malaria or bacterial infections [5,30,32].
**Hemoglobin—measured by Hemocue Hb 801 (Hemocue AB, Ängelholm, Sweden)**
*Danger sign*: severe anemia (<5 g/dL) [5]
*Procedure*: SOP in Supplement S7
*Clinical implications*: Rapid and bed-side measurement on capillary blood is important for identification of children who require urgent blood transfusion [5] and for volume adaptation of venous blood samples for further diagnostic work-up to prevent iatrogenic anemia [35]. Severe anemia is a diagnostic clue pointing to severe malaria and/or invasive non-typhoidal *Salmonella* bloodstream infections [36,37].

### 2.4. Selection of Devices: General and Study-Specific Criteria

For selection of devices and guidance in the procurement process, we consulted the WHO List of Essential Diagnostics [26], the WHO Interagency List of Priority Medical Devices for child health [23], the WHO technical specification sheets [24], the UNITAID fever diagnostic technology landscape paper [2] and the UNICEF Supply Catalogue [57].

We adhered to general criteria for point-of-care handheld devices in low-resource settings, i.e., they must be affordable, accurate, robust, rapid and user-friendly [2,4,29]. In addition, we had specific criteria related to the study setting and the capacity building component: devices must be standalone, i.e., without the need for integration in complex monitoring systems [2,4,29], they must be bedside-operated and provide results within 5 min, be robust to environmental conditions (temperature, humidity, dust) and battery powered to allow use during power cuts. For the instructions for use, a printed format in French language was preferred. Affordability must comprise total cost of ownership at retail prices, delivery time should be maximum three months.

### 2.5. Procurement and Shipment of Handheld Diagnostic Devices

All devices were ordered and procured in Belgium and shipped in batch to Kisantu Hospital. All correspondence was done by the English-proficient principal investigator of the DeNTS study (B.T., pediatric resident) supported by a research team competent in in vitro diagnostics.

### 2.6. Training, Use and Maintenance of Handheld Diagnostic Devices

Instructions for use (IFU) were integrated into French language standard operating procedures (SOPs) by the principal investigator. Device-specific training of end-users at study initiation was based on these SOPs and organized by the principal investigator. Both IFU and SOPs were at the disposal of all end-users at the site of triage. After training, end-users were supervised by the principal investigator on-site during the first 6 weeks of the study. Hereafter, supervision was organized remotely by weekly teleconferences and ad hoc needs-based contacts (email, WhatsApp), and as part of on-site supervisory visits. During the first 4 weeks of the study, a rotation system was used to ensure that all end-users got familiar with all devices. After these 4 weeks, each healthcare worker was assigned specific responsibilities to create one expert per study task. Nevertheless, all end-users continued to use all devices during weekend duties or moments of busy patient flow.

### 2.7. Evaluation of Instructions for Use

We evaluated the accessibility (format, language) of IFU of the implemented handheld diagnostic devices. In addition, we assessed readability of the IFU instructions for (preparations of) the measurement with an online tool that determined the educational level needed to understand a French language text [58]. Finally, clarity and integration of figures of the IFU were evaluated.

### 2.8. Usability Assessment and Label Comprehension Study

After 10 months of use, we assessed the usability of the devices based on a semi-structured group interview (Supplement S2A) with eight end-users, among whom six research nurses and two research physicians. Questions of the semi-structured interview were based on guidance from the Food and Drug Administration [59], the Scandinavian evaluation of laboratory equipment for point of care testing (SKUP) and inspired by previous usability studies with hemoglobinometers [60,61] and pulse oximeters [38,62]. Questions were initially written in English and translated to French. Finally, we organized a short label comprehension study in which seven end-users were asked to write down the meaning of a selection of 20 labels (Supplement S2B).

## 3. Results

We used four handheld diagnostic devices (Figure 3 and Figure 4), i.e., a tympanic thermometer (Genius 3, Cardinal Health, Dublin, OH, USA), a multimodal pulse oximeter that measured oxygen saturation, heart rate and respiratory rate (Rad G continuous pulse oximeter, Masimo, Irvine, CA, USA), a blood glucose meter (glucometer, Accu-Chek Performa, Roche Diagnostics, Mannheim, Germany) and a blood hemoglobin meter (hemoglobinometer, Hb 801, Hemocue AB, Ängelholm, Sweden).

### 3.1. Device Selection: Insufficient Guidance

WHO only provided detailed technical specifications for tympanic thermometers [17]. For the other devices, WHO guidance documents were generic and limited to the type of device required per level of care [16,19], e.g., a glucometer for capillary whole blood measurement at health-facility level without laboratory [19]. The UNICEF supply catalogue provided target product profiles and made a selection of useful devices with their respective technical specifications [23,41,46]. The UNITAID fever diagnostic technology landscape paper recommended devices based on their technical profile, e.g., recommendation of Masimo Rad G pulse oximeter as a multimodal device [2]. Therefore, we based our selection of devices more on the latter two sources than on WHO guidance. We also took into account independent product evaluations of currently marketed devices [29,30,31,32,35,36,37,38,39,40,42,43,44,45,47].

### 3.2. Procurement: Insufficient Affordability, Delivery and Access

Three devices were initially selected, but finally not ordered due to COVID-19 related stock rupture (ThermoScan 5 tympanic thermometer, Kaz USA, Southborough, MA, USA), high cost of ownership (Masimo Pronto/Rad-67 transcutaneous hemoglobin meter, Masimo, Irvine, OH, USA: USD 1–2 per test) and market withdrawal due to a programming error (ChARM respiratory rate counter, Philips India Ltd., Kolkata, India). Although this market withdrawal had preceded the order, the distributor accepted the order. The distributor (the only distributor of the device worldwide) required a minimum order of 20 pieces.

### 3.3. Training, Use and Adoption: Robustness, Timely Results, Ease of Sampling and Client-Centeredness

Environmental temperature sometimes exceeded the maximum operating temperature (33 °C) of the tympanic thermometer causing non-function and a need to cool-down the thermometer. Overheating particularly occurred when the thermometer was transported in the pocket of a healthcare worker’s uniform, but also when transported in a separate, non-covered box. The multimodal oximeter failed to deliver results in-time with 6% (12/202) measurements lasting more than 5 min (see previous study [21]).

Difficulties in capillary blood sampling and transfer of blood to strips and microcuvettes caused unreliable glucose and hemoglobin measurements at the start of the study. Poor repeatability of hemoglobin measurements (up to 2 g/dl differences) was linked with subtle underfilling of the microcuvette and “milking” of the finger during capillary blood sampling. Incorrect detection of hyperglycemia (Figure 4) was most probably caused by sugar residues on the child’s finger that were incompletely removed as washing hands with water and soap (recommended in the IFU) was not possible due to the absence of running water at the triage.

**Figure 5:**
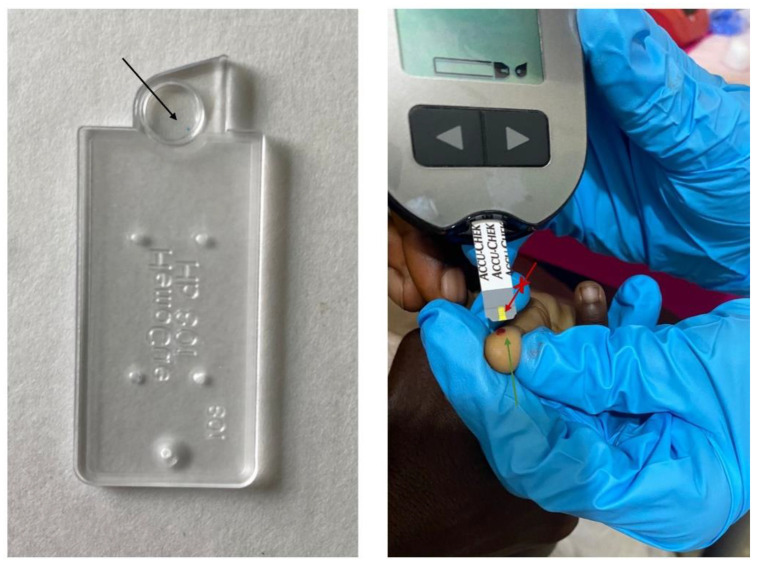
Pictures illustrating the blue dot observed in the microcuvettes of the hemoglobinometer (black arrow) and application of blood to the front edge (green arrow) and not on the top (red arrow) of the glucometer strip.

Blue dots in the hemoglobinometer’s microcuvettes were observed (Figure 5), which was worrisome because precision of the hemoglobinometer was initially poor. Our demand of technical support was immediately replied by the manufacturer, but they requested a detailed report comprising technical but also clinical information including patient characteristics, diagnosis, treatment, outcome and individual clinical decision making of the concerned patients as well as detailed procedures of routine patient management. An intense and lengthy email correspondence with the principal investigator was started and oriented towards liability and potential user errors. A final answer explaining that the blue dot was evaporated reagent that did not interfere with the analysis was provided 6 months later.

### 3.4. Instructions for Use: Insufficiently Clear and Not Adapted to Low-Resource Setting

French language IFUs in a printed booklet format were only available for the hemoglobinometer and glucometer (Table 1). In the IFU of the control fluid for the hemoglobinometer (HemoTrol Duo control fluid Low (Ref AN01637A01) and Normal (Ref AN01637A02), Eurotrol, Ede, The Netherlands), an error in the French language translation was observed (shelf-life of 14 days instead of 31 days after opening). All four devices had a readability score at the primary school level and contained a symbol key table explaining the meaning of the symbols.

For the multimodal oximeter, IFUs from the oximeter and sensor were separate documents. As a consequence, no complete, step-by-step instructions for connecting, measuring and reading was available. In addition, figures on the use of the sensor were not integrated in the text. For the tympanic thermometer, the figures did not provide guidance on the alignment of the probe with the auditory canal, which is the most difficult step of the measurement.

For the hemoglobinometer and the capillary glucometer, the IFU sampling procedure did not provide alternatives for cleaning the finger prick site when running water was not available on site, as was the case in our setting. The IFUs of both devices did not provide information about the optimal order to take samples from a single capillary finger prick in case of multiple testing (malaria rapid diagnostic test, glucose and hemoglobin measurement, Figure 4).

### 3.5. Maintenance: Cold-Chain and Environmental Conditions Challenge Quality Control and Calibration

The tympanic thermometer needed twice-yearly calibration but this was not clearly communicated at procurement. On-site calibration was not feasible due to narrow operational temperature of the calibration device while the calibration service offered by the manufacturer could not be used due to the absence of a local distributor. Quality control of the hemoglobinometer was challenged by the cold chain transport requirements of the control fluid which needed to be imported.

The usability evaluation (see below) learned that the end-users considered maintenance and cleaning as feasible for all devices, although cleaning of electronic or measurement parts (e.g., of the hemoglobinometer) was reported as delicate. End-users declared a preference for a rechargeable system instead of batteries for the glucometer and hemoglobinometer. In case of the thermometer, a screwdriver was needed to change the batteries. Quality control for the glucometer was declared complex and confusing, in contrast to the quality control for the hemoglobinometer which was considered as relatively easy.

### 3.6. Usability Evaluation: Detailed Feedback and Concrete Suggestions for Device Improvement

The label comprehension study revealed very limited comprehension of the meaning of the symbols labeled on the devices, their package and IFU. For 6, 6 and 3 out of 20 symbols, there were 0, 1 and 2 correct answers, respectively; median correct scores for nurses and physicians were 4/20, and 8/20, respectively (Supplement S2B).

A detailed interview from all feedback and suggestions made during the usability interview is described Table 2 and Table 3. End-users declared that they were initially non-confident to use all four devices but gained confidence with practicing the thermometer and glucometer. Correct filling of the hemoglobinometer’s cuvettes remained a challenge; only the nurse who daily used the hemoglobinometer declared she felt fully confident to do so. Skin cleaning for the glucometer was perceived as “annoying”, due to the need for triple antisepsis with 30 s waiting time to let the alcohol evaporate between every disinfection step in the absence of running water. End-users also reported difficulties to apply the blood at the front edge of the glucometer strip instead of on top of the strip (Figure 5) and pointed to the lack of harmonized glucometer strips.

All end-users, including the one who used it daily, reported that they struggled with the multimodal oximeter, particularly because of the occasionally long measurement times for the respiratory rate which disrupted patient flow at the triage. By contrast, they highly appreciated the short time-to-result of the thermometer, glucometer and hemoglobinometer. For these three devices, they also liked the memory function and large display of the results with someone clarifying that she “can even read the results without glasses”. End-users appreciated the self-explanatory error messages of the thermometer but struggled with the alphanumeric coded error messages of the glucometer and hemoglobinometer.

End-users were in favor of the size and weight of the multimodal oximeter, glucometer and hemoglobinometer. They stated that the thermometer was relatively bulky and that they “had thought it would have additional functions given its size”. End-users further noted that the thermometer probes and oximeter sensor were not adjusted to the size of the smallest children. They also struggled with the single-use probes of the thermometer, because these were sometimes difficult to attach swiftly and impractical as they had to carry along a small waste bin to throw them away.

Use of the multimodal oximeter to measure the oxygen saturation and heart rate was reported not difficult when children were calm. However, many children were agitated or got agitated during the measurement. Agitation complicated measurements by the multimodal oximeter, correct positioning of the thermometer and capillary blood sampling for the glucometer and hemoglobinometer. The interviewed staff further reported that the alarm sounds of the multimodal oximeter worsened agitation of the child and alerted the children’s caretakers who associated the oximeter with severe illness and poor prognosis. By contrast, some children got used to the thermometer during hospital follow-up and perceived it as a telephone toy.

Overall, when asked if they would buy the device again irrespective of affordability, end-users reported that “they would pay a lot of money for the hemoglobinometer”. They would also buy the glucometer, but not the multimodal oximeter. They would buy the thermometer if there was a solution for the single use probes.

## 4. Discussion

### 4.1. Summary of Findings

In a field study addressing triage of children under five with severe febrile illness in a rural district hospital in sub-Saharan Africa, we used handheld diagnostic devices to guide detection of those in need of hospital admission and urgent antimicrobial and supportive treatment. Three devices were selected, but stock rupture, market withdrawal and unaffordable cost precluded their procurement. The four selected, procured and used devices (tympanic thermometer, multimodal oximeter, glucometer and hemoglobinometer) had challenges and shortcomings along their lifecycle, compromising fulfilment of the REASSURED-criteria [27].

### 4.2. Device Selection Needs Centralized and Comprehensive Target Product Profiles

WHO guidance documents were generic (list of devices recommended per level of care) and not detailed. A few target product profiles compiled by other international actors and some independent device evaluations were retrieved, but information was scattered and fragmented. Target product profiles and technical specification sheets should be centralized and cross-referenced among international actors. In addition to guiding selection, independent product evaluations are valuable to understand performance and limitations. Although we did not retrieve independent product evaluations in the reference section of the IFUs assessed, they have certainly a place there [63].

### 4.3. Procurement and Shipment Need for Transparent Communication and In-Country Distributors

The transcutaneous hemoglobinometer was very attractive for triage given its non-invasiveness and potential speed, but we did not procure it because of a high fixed price (2 USD) per test, exceeding the acceptable (“minimal”) and desirable (“optimal”) price per test cited by the UNICEF hemoglobinometer target product profile for low-resource settings (0.50 and 0.05 USD, respectively [55]). The fixed price per test was not related to consumable or equipment cost but set by fixing a limited number of measurements in the sensor. Profit-oriented sales strategy was also observed for the (non-procured) ChARM respiratory rate counters for which minimal order of 20 devices was asked.

Based on present procurement experiences, we recommend that product-cycle oriented information is shared upfront and easily accessible online including a certificate of regulation, needs for calibration and maintenance and a list of common accessories and spare-parts. International shipments considerably increased total cost of ownership and complicated quality control (cold chain requirements of control fluid for hemoglobinometer) and calibration (twice yearly calibration of tympanic thermometer in a Belgian hospital). Moreover, the fact that the distributor of ChARM respiratory rate counters was unaware of their withdrawal from market suggests poor communication with the manufacturer. National diagnostic strategies and governance have the potential to improve in-country supply [4,64].

### 4.4. Training and Adoption Need Prioritization and Clear and Accessible Instructions for Use

The device implementation phase required high investments (SOPs, hands-on training workshops, supervised rotation, “device experts”). This means that, when implementing such devices in a routine frontline diagnostic setting, prioritization and gradual adoption is key. The study staff prioritized the glucometer and hemoglobinometer, which provide actionable results and, in case of the glucometer, provides information that cannot be generated from the clinical exam.

Not all IFUs were available in a printed French-language format and in the case of the tympanic thermometer and in particular the multimodal oximeter, figures and components could be better integrated and aligned with the written instructions. Readability of instructions for use was excellent, but was only assessed for the measurement instructions, which is the easiest and best structured section of instructions for use. The actual readability level of the procedure’s section of the IFU (for all devices estimated at primary school level) represents the target for non-laboratory trained staff [48] but is appropriate since the procedure’s section is the most operation of the IFU and since French is the official language but not mother tongue of most healthcare staff on site.

End-users preferred error messages depicted as graphical symbols rather than as alphanumeric codes and this apparently contrasted with the low scores in the label comprehension study. The low scores may partially be explained by the stand-alone versus contextual presentation of the symbols but comprehension of internationally recognized symbols is generally poor to moderate among non-trained participants [65,66]. Symbols however transcend language barriers and stand out for their high visual impact and noticeability. Given their synergy with textual instructions, their use and message should be actively promoted, for instance by adding a symbol key table to the IFUs [63], as was the case for all four devices evaluated.

### 4.5. Usability and Performance Need Ease of Specimen Collection

Pivotal to usability and performance was the ease of specimen collection, which is particularly important during triage by healthcare workers with limited clinical expertise [2,27]. Present challenges included alternatives for skin cleaning in the absence of running water and the order-of-draw of successive tests performed on capillary blood. Given the increasing panel of bedside point-of-care diagnostics [67], single-product performance studies should be complemented with field studies addressing multiple parameters and co-endemic diseases in low-resource settings [64,68,69,70]. Ease of specimen collection might also benefit from engineering facilitating correct filling of the hemoglobinometer’s microcuvettes and resolve the counterintuitive filling of the glucometer strips. Likewise, smaller size tympanic thermometer probes and multimodal oximeter sensors can improve measurements among the youngest and smallest children.

### 4.6. Usability: Robustness, Size, Time-to-Result, Easy Reading, Perception by Patients and Caretakers

Failure of the thermometer in a too-hot environment confirmed the importance of robustness. Further, they appreciated the handy glucometer but disliked the bulky tympanic thermometer; they liked a large display of all devices the presence of a memory function and except the multimodal oximeter. Large font-sizes are particularly important in a low-resource setting, where impaired vision is often undercorrected [71,72]. The need for speed was confirmed by the disruption of patient flow linked to the slow performance of the respiratory rate counting by the multimodal oximeter.

Of note, the usability interview revealed the importance of patients’ and caretakers’ experiences and perception. Children were afraid or became agitated by the long measurement and alarm sounds of the multimodal oximeter, which also caused unrest among caretakers, hampering also the other measurements. Moreover, caretakers perceived the multimodal oximeter as a sign of bad prognosis. These observations show the importance of assessing perceptions in the field-context of the intended use and user.

### 4.7. Usability Needs Local, Client-Centered Technical Support and Human Factor Engineering

A consistent point impacting affordability, delivery and access was the lack of an in-country distributor, which required logistics (stock management, international shipments), impacted work (calibration) and entailed additional costs. In-country technical support services are highly recommended as they could also assist with on-the-job-training, appropriate use and integration in daily practice, calibration, quality control, maintenance and disposal [7].

Correspondence with remote manufacturers occasionally caused misunderstanding. Upon a technical question (presence of blue dots in the hemoglobinometer’s microcuvettes), the manufacturer replied with a request for extensive and detailed technical and clinical information. Although sufficient detail is required for sound investigation of customers’ complaints and post-market surveillance [21], the level of detail and subsequent orientation towards liability and potential errors at the study site were perceived as not related to the question raised and not adapted to the end-user’s professional and sociocultural context. Complaint handling, let alone correspondence between manufacturers and end-user, should occur in a safe climate and errors should be considered as use errors preventable by human factor engineering [73]. Mutual and regular exchanges between manufacturers and end-users as well as the presence of local distributors may prevent communication mismatches.

### 4.8. Maintenance, Repair and Disposal: Equipment-Free and Environment-Friendly

As discussed above, quality control of the hemoglobinometer and calibration of the thermometer were challenged at product selection (clear upfront communication of requirements) and by issues of access and delivery (no local distributor, cold-chain requirements, operating temperature). All devices except the multimodal oximeter had single-use consumables (strips, microcuvettes and probes) requiring disposal and challenging environment-friendliness. End-users declared a preference for rechargeable integrated batteries (provided enough autonomy, universal adaptor) which facilitate stock management and decreases the inherent disadvantages if replaceable batteries (poor performance, leakage, theft, needs for utensils, disposal, costs).

### 4.9. Limitations and Strengths and Generalizability

A major limitation of this study was its retrospective design. However, the study was embedded in a prospective clinical study (DeNTS study) commanded by the same principal investigator and study team. All incidents and associated decisions were documented as part of the DeNTS study, i.e., in preparatory communication during selection and procurement processes, SOPs, weekly reports, log sheets, case report forms etc. The perspective of the end-users might have been biased by the fact that they were part of a study team, which was in-depth trained and monitored. However, study nurses and physicians had similar qualification, background knowledge and experience as the other hospital staff. They were working in the same busy triage setting and focused on patient care rather than on device evaluation per se.

As to generalizability, the present findings cannot as such be extrapolated to the health center level, where frontline healthcare workers are often less skilled and experienced [2,74], which might further complicate usability. By contrast, the hospital setting and the DeNTS study provided a high proportion of children with danger signs and confirmed invasive bacterial infections [28] and are representative for many settings in sub-Saharan Africa. Of note, danger signs are generally more difficult to assess in children compared to adults [2]. We therefore believe that the end-user as well as the study setting were a good proxy for the intended triage setting and representative for the real-life conditions and usability in sub-Saharan Africa.

The assessment of usability, IFU and label comprehension in combination with reported field incidents allowed comprehensive evaluation. Little guidance is available on how to assess usability in low-resource settings but the semi-structured interview allowed detailed feedback and revealed multiple suggestions for device improvement. We did, however, not address sensitivity or specificity, which are key determinants of appropriate and effective devices.

### 4.10. The Broader Context of Medical Devices in Low-Resource Settings and the Way Forward

Screening and diagnosis are the bottleneck of appropriate patient care [4] and nearly half (47%) of the global population does not have access to diagnostics, particularly in low-resource settings [4]. Ensuring equitable diagnostic access requires sustained prioritization, commitment and investment [4]. Adoption of the handheld diagnostic devices presently studied in the national essential diagnostic lists would allow harmonization, bulk procurement and price setting [64]. Pending sound implementation of regional and national regulations in low resource settings, diagnostic devices could benefit from an alternative quality umbrella such as WHO prequalification [75] or a similar process. Manufacturers would benefit from a stable and predictable market, which would attract local distributors and client-centered technical support services. They would also get familiar with low-resource settings and have incentives to involve end-users and national regulatory authorities as important pre-market, market and post-market stakeholders [4]. Finally, to fully exploit stakeholder involvement, end-users should be aware and mobilized to interact with authorities and manufacturers.

## Figures and Tables

**Table 1:** Evaluation of instructions for use of handheld diagnostic devices used for hospital triage of children under the age of five with severe febrile illness. Abbreviations: IFU: instructions for use; EN: English; FR: French; NL: Dutch; oth.: other languages.

	Tympanic Thermometer	Multimodal Oximeter	Glucometer	Hemoglobinometer
**Format and languages**	Digital: CD-ROM, online PDF (EN, FR, NL, oth.)	Oximeter: Printed (NL), Digital: online PDF (EN) Sensor: Printed (EN, FR, oth.)	Printed and digital: online PDF (EN, FR, NL, oth.)	Printed and digital: online PDF (EN, FR, NL)
**Readability** score (in brackets) of French sampling instructions	Primary school level (60)	Based on IFU sensor only:Primary school level (66)	Primary school level (52)	Primary school level (51)
**Symbol Key** (explanation of symbols used)	Present	Present	Present	Present
**Figures**	Could be more detailed to better represent wording IFU Corresponded to reality	Position of sensor not sufficiently detailed and not clearly indicated which picture corresponds to which age category Figures and text not integrated into a stepwise illustrated procedure	Clear Corresponded to reality and wording IFU	Clear, real-life colors Corresponded to reality and wording IFU
**Comments from end-users** to adapt sampling instructions to real life situations in low-resource settings	Visual inspection of auditory canal should be more detailed, i.e., describe as a separate step what should be looked for Detailed figure on alignment of the probe with the auditory canal is missing	No step-by-step IFU: set up and features of device are described in IFU of oximeter, while site selection and sensor application are described in IFU of sensor A few inconsistencies in the wording used in IFU sensor, e.g., weight, hearing a tactile click	No instructions on how to clean skin in the absence of running water No instructions on how to integrate in multisampling procedure for multiple rapid diagnostic tests from a single finger prick	Importance of correct sampling highlighted, but no detailed anticipation of what can go wrong, e.g., no “milking” or massing of finger, or how complete filling can be verified No instructions on how to integrate in sampling procedure for multiple rapid diagnostic tests from a single finger prick

**Table 2:** Usability evaluation based on a semi-structured interview of end-users along the lifecycle of handheld diagnostic devices to measure tympanic temperature and respiratory rate. Legend: HCW: healthcare workers, caretaker: parent or other person taking care of the child upon admission.

	Tympanic Thermometer	Multimodal Oximeter
**Frequency of use** (numbers refer to study HCW: n = 8)	Daily: n = 8	Daily: n = 1 2 – 4 days/month: n = 5 < 1 day/month: n = 2
**Training & adoption**	🗸 Rapidity facilitates patient flow 🗶 Difficult to learn correct position 🗶 Large size hampers integration in routine care 🗶 Non-function if environmental temperature is too high hampers integration in routine care	🗶 Thorough training & practice is needed. 🗶 Difficult to know when & how to change sensitivity mode 🗶 Difficult to integrate in routine care due to slow performance & unreliable respiratory rate: *“It makes the user suffer”*
**Measurement preparation**	🗸 Few steps & rapid 🗸 Ear is an easily accessible body site 🗸 Difficult access if child moves or is afraid 🗶 Sometimes difficult to take up probes from base & attach them to the thermometer 🗶 Inspection of ear canal needed	🗸 Few steps & rapid if child is calm 🗶 Some children refuse the sensor application & it can take long to calm the child: *“You sometimes have to wait until the child sleeps.”*
**Measurement**	🗸 Rapid 🗸 Easy, few steps 🗸 *“Does not miss subjective fever”* 🗶 Thermometer shuts down if you are too slow 🗶 Not reliable (too low) if not well positioned, if child moves, or if ear canal is wet because the child is sweety or freshly washed 🗶 Impression that the device displays the previous temperature if measurement interval between two consecutive measurements is too short, although probe was changed	🗸 Measurement of oxygen saturation and heart rate is timely and reliable if child is calm 🗶 Measurement of oxygen saturation and heart rate take time & some- times multiple attempts needed. The plethysmography curve is irregu- lar if the child moves too much 🗶 Measurement can be so slow that a child that was initially calm gets agitated during measurement 🗶 Respiratory rate measurement is unreliable & slow
**Result display**	🗸 Easy to read 🗸 Rapid 🗸 Memory function	🗸 Good readability of numbers 🗸 Plethysmography curve displayed 🗶 Preliminary result displays in grey, this is not described in IFU. It can take long until definitive measurement displays in white. 🗶 If measurement is unsuccessful “- -“ is displayed. There is no error message explaining the error source. 🗶 Continuous measurement: you must be quick to read measurement be- fore sensor disconnection & there is no memory function
**Maintenance**	🗸 Easy cleaning 🗶 Need for screwdriver to change batteries	🗸 Easy cleaning 🗸 Rechargeable with USB-cable 🗶 Careful cleaning of electronic connection between sensor & oximeter
**Quality control & calibration**	🗶 Need for frequent recalibration 🗶 No recalibration on site possible	🗸 No need for recalibration 🗶 No external quality control possible
**Hygiene & security for HCW**	🗸 No need to touch used probes (eject button) 🗶 Need to carry along a small waste bin to throw away used probes	🗸 Feel comfortable during use
**Security and comfort for patient & caretaker**	🗸 Parents trust the device 🗸 Considered as a play *(“telephone*”) by some children 🗶 Other children are afraid	🗸 Non-invasive technique 🗸 High-tech interface is considered as high-quality care 🗶 Caretakers associate the use of an oximeter with the presence of severe illness & think that it can predict disease prognosis 🗶 The alarm sounds scare the caretakers and children: *“Children are really afraid, it’s a war.”*
**Size & bedside testing**	🗸 Rapid 🗶 Too big and heavy 🗶 Size hampers integration in routine care 🗶 Probe is too large for ears of small children	🗸 Not heavy 🗶 Sensor is too large for smallest infants
**Suggestions**	> Reduce size & weight > Reusable probes, cleaning instead of probes > Enable on site recalibration	> Spot-check mode with memory function (available on market, was not deliverable upon order) > If not improved, omit respiratory rate measurement > Integrate screen in sensor to make device smaller > Improve function in severely ill children > Provide a smaller sensor for the smallest infants > Add an indicator led light that illuminates when the device is charging

**Table 3:** Usability evaluation based on a semi-structured interview of end-users along the lifecycle of handheld diagnostic devices to measure blood glucose and hemoglobin. Legend: HCW: healthcare workers, IFU: instructions for use, caretaker: parent or other person taking care of the child upon admission.

x	Glucometer	Hemoglobinometer
**Frequency of use**	Daily: n = 1 2 – 4 days/month: n = 5 < 1 day/month: n = 2	Daily: n = 1 2 – 4 days/month: n = 5 < 1 day/month: n = 2
**Training & adoption**	🗸 For most staff: easy & quick learning 🗸 Rapidity facilitates patient flow 🗶 For less experienced nurses: initially a bit complex to remem- ber & organize all different steps	🗸 Rapidity facilitates patient flow 🗸 Not difficult to learn which steps must be done 🗶 Correct sampling requires practice & experience 🗶 Confusion in the first weeks of use due to variable reliability of results compared to results routine lab. Improved after new sampling instructions. Trust in Hemocue Hb801 restored after on-site comparison with routine hematocrit values
**Measurement preparation**	🗶 Cleaning of skin is difficult because fingers are often very dirty & running water is not available. It takes min. 3 disinfection steps to clean the skin. 🗶 If cleaning is not properly done, sugar residues on the skin cause falsely high glucose measurements 🗶 Slow: Waiting until disinfectant completely dries & waiting until device is ready for blood application after strip insertion 🗶 Strip must be correctly oriented upon insertion. If not: coded error message appears	🗸 Easy to prepare measurement 🗶 If device activated too soon, it returns to sleeping mode be- fore microcuvette is filled & ready for insertion
**Measurement**	🗸 Few steps required, rapid 🗸Blood automatically aspired in strip upon contact 🗶 Entry point for blood aspiration is small and it is counterintui- tive & impractical that it is located at the front edge, instead of on top of the strip, particularly difficult if child moves a lot 🗶 Blood glucose is last test that is sampled from the finger prick. Sometimes a 2^nd^ finger prick is needed because meanwhile the blood stopped flowing	🗸 Few steps required, rapid 🗸 Blood automatically aspired in strip upon contact 🗶 Difficult to fill microcuvette correctly/completely: if child moves, if blood drop is small, if drop is poorly delineated (particularly in severely anemic children). 🗶 Massing finger or strong pressure on finger can disturb result
**Result display**	🗸 Easy to read 🗸 Rapid 🗸 Memory function 🗶 Error messages are not self-explanatory	🗸 Easy to read 🗸 Rapid 🗸 Memory function 🗶 Error messages are not self-explanatory
**Maintenance**	🗸 Easy cleaning 🗸 Easy to change battery 🗶 *“Once, I accidently entered the set-up mode & could not use the de vice anymore until the principal investigator explained me how to leave the set-up mode.”*	🗸 Easy to change batteries 🗶 Cleaning is a bit delicate & complicated: concentration needed to clean the interior part without leaving cotton particles; wait until all parts are dry before reassembling; sometimes difficult to reinsert the microcuvette support 🗶 Once we suddenly could not turn on device anymore: AAA batteries corroded. Problem resolved by changing batteries
**Quality control & calibration**	🗸 The quality control has always been within range. 🗶 Quality control is very complex: many steps, *“sometimes you must click once & sometimes twice”*, need to consult the proce- dure because failure to remember all the steps. 🗶 The 2 different control liquids are easily confused.	🗸 Quality control is easy & has always been within range 🗶 Biosecurity risk because quality control fluid is blood 🗶 Quality control fluid is expensive and difficult to get and store due to cold chain requirements
**Hygiene & security for HCW**	🗸 Small blood volumes increase safety. 🗸 All staff feels comfortable during the procedure.	🗸 All staff feels comfortable during the procedure 🗶 A large drop of blood is needed: there is a risk that the blood starts flowing if the drop gets too large
**Security and comfort for patient & caretaker**	🗸 Caretakers aware of importance of glycemia for good health: glycemia measurement highly appreciated by caretakers 🗶 Children are afraid from the finger prick and cry. Caretakers are sometimes afraid too 🗶 Caretakers worried if measurement must be repeated when glycemia possibly falsely elevated due to sugar residues on child’s hands	🗸 Caretakers want to know if their child is anemic and if their child needs a transfusion 🗸 Caretakers are not worried about the measurement 🗸 Caretakers are very happy with the instant results 🗶 Children are afraid from the finger prick and cry Caretakers are sometimes afraid too
**Size & bedside testing**	🗸 Small & not heavy, fits in your pocket 🗶 Single use strips 🗶 Strips not universal for all glucometers	🗸 Small & not heavy 🗸 Easy to carry around
**Suggestions**	> Universal strips > Change to rechargeable device > Switch to non-coded, self-explanatory error messages > Simplify blood sampling: on top instead of edge strip > Appropriate disinfectant / cleaning solution	> Opt for rechargeable battery (already available on market) instead of AA battery (used in current study) > Switch to non-coded, self-explanatory error messages > Facilitate correct use by clear sampling instructions or training support

## Data Availability

No new data were created or analyzed in this study. Data sharing is not applicable to this article.

## Supplementary Materials

The following are available online at https://www.mdpi.com/article/10.3390/diagnostics12030746/s1. **Supplement S1.** WHO recommendations concerning the life cycle of the handheld diagnostic devices, from development to disposal: extended version, **Supplementary Figure S1.** Key elements in the lifecycle of handheld diagnostic devices at the health-facility level, based on the WHO Technical Series on Medical Devices to ensure improved access, quality and use of medical products and technology. Reference [76] is cited in the supplementary materials. **Supplement S2.** Semi-structured usability interview and label comprehension study. **Supplement S3.** Standard operating procedure tympanic thermometer. **Supplement S4.** Review document infrared thermometry and fever cutoffs. **Supplement S5.** Standard operating procedure multimodal oximeter. **Supplement S6.** Standard operating procedure glucometer. **Supplement S7.** Standard operating procedure hemoglobinometer
