# Supplementary material for: Field Experiences with Handheld Diagnostic Devices to Triage Children under Five Presenting with Severe Febrile Illness in a District Hospital in DR Congo
Source: Diagnostics (Basel). 2022 Mar 18;12(3):746. doi: 10.3390/diagnostics12030746 (PMC8947034; doi:10.3390/diagnostics12030746)
Supplement: Supplementary file 1 [file diagnostics-12-00746-s001.zip › Supplement Proofs/220104_BT_Field experiences_S3.pdf]

	**Titre:** Mesurer la température auriculaire avec Genius 3<br>**Premier auteur:** Bieke Tack<br>**Dernière révision:** 26/11/2020
	**Etude HIT BSI:** Health itinerary of young children with suspected bloodstream infection in Kisantu, DR Congo: a cohort study<br>**Etude DeNTS:** Clinical decision support in non-typhoidal *Salmonella* bloodstream infections in children in sub-Saharan Africa: a prospective cohort study<br>**Etude TreNTS:** Treatment of non-typhoidal Salmonella bloodstream infections in children in sub-Saharan Africa: a prospective cohort study

1. Domaine et application

Ce document fournit les instructions pour mesurer la température auriculaire avec le thermomètre Genius 3 (Covidien), un thermomètre tympanique infrarouge.

2. Responsabilité

Fonction	Activités
Infirmier(e) ou Médecin	• mesure la température<br>• enregistre les résultats dans le cahier des observations (« CRF »)

3. Procédure

3.1 Préparation - Matériel requis

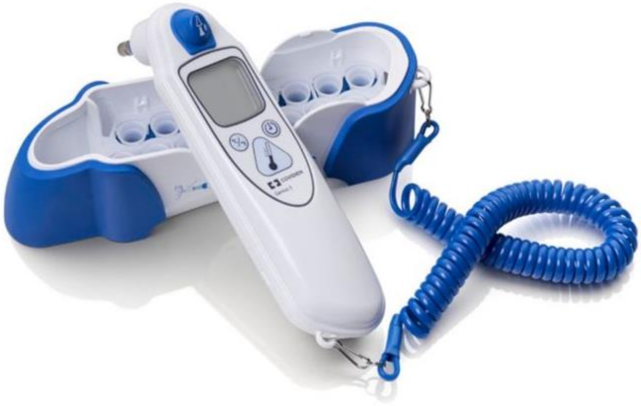

- Thermomètre Genius 3
  - Plage de mesure : 33 – 42°C
  - Stockage: température entre -25 et 55°C, humidité ≤90%
  - Conditions de mesure: température ambiante entre 16 – 33°C, humidité entre 15 et 90%, à une distance de séparation minimale de 0.6m de tablettes/téléphones
  - Alimentation: 3 piles alcalines AAA, regardez dans le mode d'emploi pour voir comment remplacer les piles
- Protections de sonde à usage unique :
  Utilisez seulement les protections de sonde de la marque Covidien !
- Verificateur/calibrateur de thermomètre
  - Plage de mesure : 32.22 ± 0.3 – 40.55 ± 0.3 °C
  - Stockage: température entre -25 et 55°C, humidité 85%
  - Conditions de fonction: température ambiante entre 21.1 – 26.7°C, humidité entre 30 et 70% sans condensation
  - Alimentation: 12 VCC, 2.5A

3.2 Procédure

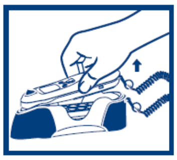	Inspectez visuellement le conduit auditif du patient. N'utilisez pas le thermomètre en présence d'écoulements d'oreille, de sang, de liquide céphalo-rachidien, des bouchons de cérumen ou des corps étrangers dans le conduit auditif.<br><br>Retirez le thermomètre de la base.
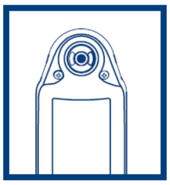	Inspectez la lentille de la sonde. Si elle présente des débris, nettoyez-la comme décrit en point 4 de cette procédure.
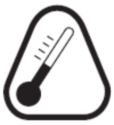<br>Bouton Balayage<br>Auriculaire<br>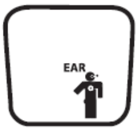	Allumez le thermomètre en appuyant sur le bouton de balayage.<br><br>Vérifiez que le thermomètre est en mode auriculaire, ce qui est indiqué dans le coin droit inférieur avec le symbole « EAR ».
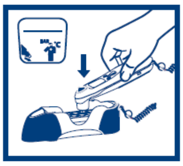 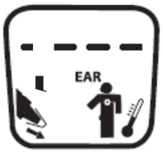	Installez une protection de sonde en poussant fermement l'extrémité de la sonde dans une protection.<br><br>Si bien fait, le thermomètre affiche des tirets, le mode d'emplacement et l'icône de l'extrémité de la sonde.
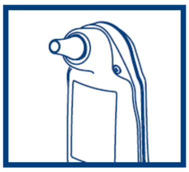	Assurez-vous que la protection de sonde est bien en place et qu'il n'y a pas d'espace entre la protection et la base de la sonde.<br><br>Assurez-vous que le film plastique ne présente pas de trous, déchirures ou plis.
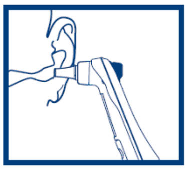 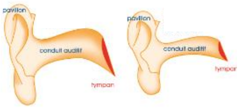 **≥ 1 an** **< 1 an**	Placez la sonde dans le canal auditif dans la position suivante :<br>– Obstruction de l'ouverture avec l'extrémité de la sonde<br>– L'axe de la sonde aligné avec le canal auditif :<br>▪ Enfant <12 mois : tirez l'oreille en arrière<br>▪ Enfant ≥ 12 mois : tirez l'oreille en haut et en arrière
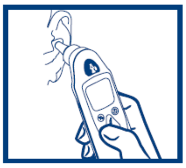 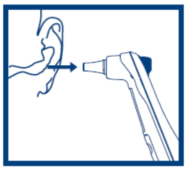	Actionnez puis relâchez le bouton de balayage.<br><br>Attendez l'émission de trois bips avant de retirer le thermomètre.
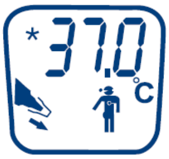	La température est affiché sur l'écran. Vérifiez que la température affichée :<br>– est exprimée en °C, sinon appuyez sur le bouton « °C/°F »<br>– est mesurée en mode auriculaire, en cas de présence du symbole « * », la température est pris en mode non-auriculaire. Si mesurée en mode non-auriculaire, changez la mode en mode auriculaire comme décrit dans le manuel d'utilisation et recommencez la procédure
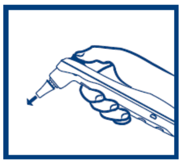 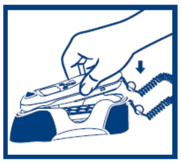	Appuyez sur le bouton d'éjection pour éjecter la protection de sonde dans un conteneur de déchets approprié.<br><br>Remettez le thermomètre sur la base après utilisation.<br><br>Enregistrez la température dans le cahier d'observations (« CRF ») du patient. Le thermomètre s'éteint 10 secondes après la prise de température. Vous pouvez consulter le dernier résultat en appuyant sur le bouton de balayage.

Remarques importantes:

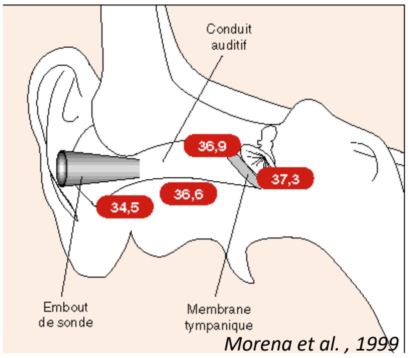

- Ne pas utiliser le thermomètre sur les patients présentant des écoulements d'oreille, du sang, du liquide céphalo-rachidien, une otite aigue moyenne, des bouchons de cérumen ou des corps étrangers dans le conduit auditif. Cela peut résulter dans des faux résultats. Dans ces cas, vous pouvez mesurer la température axillaire ou rectale avec un thermomètre digital classique. La présence de cérumen non-impacté n'affecte pas la précision.
- Il est très important de bien positionner la sonde pour ne pas avoir des faux résultats. Ceci est montré dans l'image ici à côté.
- Attendez au moins deux minutes avant de procéder à une nouvelle mesure dans la même oreille.

Interprétation de la température :

Fièvre : température de > 37.5°C
(voir « Unit review document » en annexe : TLM_RD_042_v1.0_Temperature measurement)

4. Entretien

- Protégez la lentille contre la poussière. Remettez alors toujours le thermomètre dans la base.
- Chaque jour, nettoyez et désinfectez le thermomètre :
  - Le corps, la base et le cable spiralisé :
    - Nettoyez délicatement à l'aide d'une lingette imbibé d'éthanol 70% ou alcool isopropylique 70%.
    - Désinfectez délicatement à l'aide d'une lingette imbibé d'éthanol 70% ou alcool isopropylique 70%. Maintenez une humidité visuelle pendant au moins une minute, puis laisser sécher complètement à l'air libre.
  - L'extrémité et la lentille :
    - Nettoyez délicatement à l'aide d'une lingette imbibé d'éthanol 70% ou alcool isopropylique 70%. Retirez avec précaution tous les corps étrangers de la lentille et l'extrémité de la sonde du thermomètre. Une fois les corps étrangers éliminés, séchez la lentille à l'extrémité de la sonde du thermomètre à l'aide d'un tampon non pelucheux. Pour fonctionner correctement, la lentille du thermomètre ne doit présenter aucune trace de doigts et/ou salissures.
- Ne jamais :
  - rincer le thermomètre avec un liquide vaporiser une solution nettoyante directement sur le thermomètre
  - tremper / immerger le thermomètre dans un liquide

5. Contrôle de qualité

Le thermomètre doit être vérifié et calibré au moins une fois par an (idéalement toutes les 25 semaines). La vérification et calibration du thermomètre sera fait en Belgique à UZ Leuven, si nécessaire, le thermomètre sera remplacé par un nouveau thermomètre.

Messages d'erreurs

Le thermomètre communique avec l'utilisateur à l'aide de l'écran LCD et de signaux sonores. Lorsque la protection est installée ou les piles changées, le thermomètre effectue une réinitialisation du système. Le thermomètre effectue également un autotest pour s'assurer du fonctionnement correcte.

Conditions d'alarme	Mode d'affichage	
Température du patient supérieure à la plage spécifiée	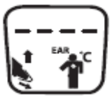	Vérifier que la protection de sonde ne présente pas de déchirures ou de trous.
Température du patient inférieure à la plage spécifiée	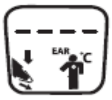	Vérifier qu'il n'y a pas de débris au niveau de la protection de sonde et de l'extrémité du thermomètre.<br>Vérifier que le canal auditif du patient ne présente pas de débris.
Température ambiante supérieure à la plage spécifiée	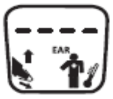	Déplacez-vous vers un environnement plus chaud / froid.<br>Laissez le thermomètre s'équilibrer à température ambiante pendant au moins 30 minutes avant utilisation.
Température ambiante inférieure à la plage spécifiée	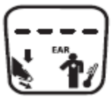	
Batterie faible	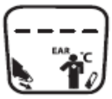	L'écran LCD affiche l'icône de pile faible. L'icône de pile faible reste à l'écran jusqu'à ce que les piles soient remplacées ou que l'indicateur de pile déchargée apparaisse. Une fois l'icône de pile faible affichée, il est possible d'effectuer environ 100 mesures.
Batterie déchargée	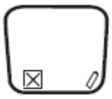	L'écran LCD affiche l'indicateur de pile déchargée. Lorsqu'un bouton est appuyé, l'indicateur clignote 3 fois puis l'écran LCD s'éteint. Une fois l'icône de pile déchargée affichée, les piles doivent être changées pour pouvoir utiliser l'appareil.

L'affichage des erreurs système « 1 » et « 2 » indique que les conditions ambiantes de la pièce sont trop instables pour utiliser l'appareil. Laisser le dispositif se stabiliser pendant 20 minutes avant de l'utiliser.

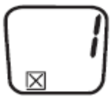

Si l'erreur système 1 est affichée, le thermomètre présente une erreur de total de contrôle au niveau de la mémoire interne (échec du test d'autodiagnostic). Installer une nouvelle protection de sonde pour réinitialiser l'appareil. Si l'erreur système persiste, prendre contact avec le centre d'entretien.

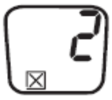

Si l'erreur système 2 est affichée, le thermomètre n'est pas étalonné (p. ex. une variable d'étalonnage se situe en dehors de l'intervalle attendu). Contacter le centre d'entretien.

Si d'autres erreurs système surviennent, installez une protection de sonde pour réinitialiser l'appareil.
Si l'erreur système persiste, contactez l'investigateur principal, ou en cas d'absence, le superviseur local ou superviseur de site.

6. Références

- Manuel d'utilisation Genius 3 thermomètre (Covidien)

7. Annexe

TLM_RD_042_v1.0_Temperature measurement

8. Histoire du document

Name and function	Date	Comments
Auteur original		
Bieke Tack	26/11/2020	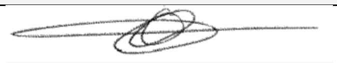
Révisé par		
Idzi Potters	11/12/2020	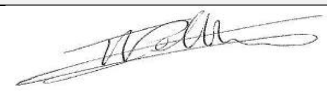
Approuvé par		
Jan Jacobs		
