# Supplementary material for: Field Experiences with Handheld Diagnostic Devices to Triage Children under Five Presenting with Severe Febrile Illness in a District Hospital in DR Congo
Source: Diagnostics (Basel). 2022 Mar 18;12(3):746. doi: 10.3390/diagnostics12030746 (PMC8947034; doi:10.3390/diagnostics12030746)
Supplement: Supplementary file 1 [file diagnostics-12-00746-s001.zip › Supplement Proofs/220104_BT_Field experiences_S4.pdf]

INSTITUTE OF TROPICAL MEDICINE ANTWERP	Department: Clinical Sciences	Unit: Tropical Bacteriology
	**Title:** Temperature measurement<br>**First author**: Bieke Tack<br>**Last Update**: 27/05/2021	
	**Project: PhD Bieke Tack:** Predicting and treating non-typhoidal *Salmonella* infections in children in sub-Saharan Africa	

Background

Fever is among the most common signs of infection. Moreover, in the context of blood culture surveillance, the presence of fever or hypothermia is a main criterium to define suspected bloodstream infection. [1,2]

The World Health Organization (WHO) and leading textbooks define [2–4]:
- **fever** as a **rectal** temperature of **> 38°C**
- **hypothermia** as a **rectal** temperature of **< 36°C**

However, rectal temperature measurement is associated with patient discomfort and emotional stress and, although rarely, with injury and transmission of bacteria . [5]

Secondly, the World Health Organization (WHO) and leading textbooks define [3,4,6,7]:
- **fever** as an **axillary** temperature of **> 37.5°C**
- **hypothermia** as an **axillary** temperature of **≤ 35.5°C**

However, axillary thermometers are slow, are prone to dislocation and to changes in ambient temperature and can be inaccurate [5,8–11].

In an attempt to perform better than oral and axillary thermometers and avoid the risks of rectal temperature measurements, non-invasive infrared thermometers have been developed. Three main types of infrared thermometers exist, *i.e.* the infrared tympanic thermometer, the temporal artery scan and the non-contact infrared skin scan. [5]

National Institute of Clinical Excellence (NICE) from the United Kingdom recommends the use of infrared tympanic thermometers for the routine work-up of a child under five years old with febrile illness.[12] In the response to the COVID-19 pandemic, the WHO recommends infrared thermometers, but does not specify which type (tympanic, temporal scan or non-contact skin scan).[13]

Aim

Provide an overview of the performance of infrared thermometers in preparation of clinical studies in low resource settings:

- to guide their use as a diagnostic criterium for blood culture sampling
- to assess body temperature and the presence of fever

This overview is not intended for:

- studies were exact temperature measurement is crucial, *e.g.* in pharmacokinetic / pharmacodynamic studies or studies with high risk decisions based on body temperature
- studies in neonates

Published literature

- We searched on PubMed using the terms “infrared therm*”, “tympanic temperature”, “non contact infrared thermomet*”, “infrared forehead thermomet*”. In addition, we searched on the websites of the following professional organizations: WHO, NICE, ESCMID, ESPID, IDSA, AAP, CDC and ECRI.
- Most retrieved studies were organized in a pediatric population.
- We focused on studies that used invasive (intracorporal) temperature or rectal temperature as a reference (ref) method.

Literature overview of three infrared thermometer types			
	**Tympanic thermometer**	**Temporal artery scan**	**Non-contact skin scan**
**Frequently used devices**	Genius (Cardinal Health)<br>ThermoScan (Braun)	TemporalScanner / TAT (Exergen)	Thermofocus 0800<br>Thermoflash
**Technique**	Contact measurement in the ear canal. To straighten the ear canal, perform an ear tug.<br>(<1 year: pull straight back,<br>≥ 1 year: pull ear up and back)	Slow scanning of the forehead in the temporal artery region and behind the ear by direct skin contact	Non-contact measurement at the forehead / temple
**Accuracy**	• **In comparison to rectal temperature, accuracy of the 3 infrared thermometers is poor:**<br>• The measured temperature can differ > 1°C from the rectal temperature [5,14–24], which is higher than the clinically acceptable difference of 0.5°C. [11,18]<br>• Most studies found a systematic difference with rectal temperature pointing towards an underestimation of the body temperature, although also overestimation occurred. [14,16–19,21,22] Three studies demonstrated an overestimation of low temperatures and an underestimation of high temperatures. [5,15,20]<br>• Tympanic thermometers performed better than temporal artery scans in 4 studies [15,18,19,22] and vice versa in 2 studies.[5,21] In 1 study, non-contact skin thermometers performed better than a tympanic, but worse than a temporal thermometer.[5]<br>• **In comparison with invasive temperature, most studies point to tympanic thermometers as accurate:**<br>• Some studies show that the tympanic thermometer was a better approximation of the temperature in the pulmonary artery (golden standard) than rectal temperature. [25]<br>• When compared to the temperature measured by an indwelling bladder catheter, tympanic measurement was better than rectal temperature. However, non-contact infrared thermometers performed worse than tympanic and rectal thermometers. [8]<br>• The accuracy of temporal artery scan when compared with invasive temperature measurements remains unclear.[23]<br>• **Infrared thermometers are more accurate than other non-invasive thermometers:**<br>• Tympanic temperature [8,10,14,24], temporal artery scan [10] and non-contact skin scan[8] showed a better performance than axillary temperature measurement when compared with rectal or other invasive temperature measurements as reference method.<br>• Tympanic temperature and temporal artery scan showed similar or better performance than oral temperature measurement when compared with rectal / pulmonary artery temperature measurement as reference method. [10,19,25]<br>• **An alternative fever cut-off for infrared tympanic thermometers has been proposed:**<br>• In line with the alternative fever cut-off for axillary temperature [3,4,6,7]<br>• Performance (sensitivity and specificity) of proposed fever cut-offs differed between studies, probably due to differences in patient population and/or devices (products).		
	**Infrared tympanic thermometer**	**Temporal artery scan**	**Non-contact skin scan**
**Standard fever cut-off = 38.0 °C**<br>(ref = rectal)	Sensitivity 68 - 92% (except 22% in [5])<br>Specificity 96 – 100%<br>[5,15,18,19,21,22,24,26,27]	Sensitivity 64 - 91%<br>(except 40% in [5])<br>Specificity 90 – 98%<br>[5,15,18,19,21,22,28]	Sensitivity 27 - 97%<br>Specificity 97 - 100%<br>[5,28,29]<br>Meta-analysis (also containing oral and axillary temperature for comparison) [35]:<br>Pooled sensitivity: 81% (95% CI: 66 – 90%)<br>Pooled specificity: 92% (95% CI: 77 – 98%)
**Alternative fever cut-off**<br>(ref = rectal)	**37.5 °C** [19,24]<br>Sensitivity 81 – 91%<br>Specificity 86 – 90%<br><br>**37.6 °C** [9] (no performance data)<br><br>**37.8 °C** [18,26]<br>Sensitivity 92 – 95%<br>Specificity 80 – 95%	**37.5 °C** [19]<br>Sensitivity 91%<br>Specificity 72%	**37.1°C** [29]<br>Sensitivity 80.8%<br>Specificity 95%
**Precision**	Acceptable repeatability [22]	Conflicting data:<br>- Acceptable repeatability [11,22]<br>- Unsatisfactory precision [23]	No data found
**Possibly distorting factors**	- Inappropriate angle to tympanic membrane [5]<br>- Ear wax [19,27], acute otitis media [5,27]<br>- Lying on the ear [19], exercise / crying [27]<br>- Age < 4 weeks - < 6 months [5,12,18,26]	No data found	Site & distance of measurement [30,31]<br>Environmental temperature [22]<br>Perspiration [10]
**Performance in tropical setting**	Similar to non-tropical setting [14,24,32]	Similar to non-tropical setting [33]	No data found

Legend table 1:

- Standard fever cut-off was based on the WHO fever cut-off for rectal temperature and defined as 38.0 °C. Alternative fever cut-offs are defined as lower fever cut-offs proposed by referred studies to increase diagnostic performance.
- Ranges of sensitivity and specificity are based on the minimum and maximum sensitivity and specificity reported in the referred studies.

Discussion

As an alternative to the invasive rectal temperature measurement and often unreliable axillary measurement, an infrared thermometer is useful. Based on the published literature, infrared tympanic thermometers outperform temporal artery scan and non-contact infrared skin scan. We did not find recommendations for use of remote infrared thermometers for infection control and prevention purposes in the healthcare settings.

We propose:

- to use **infrared tympanic thermometers** for body temperature measurement.
- to use **37.5°C:**
  - as a**lternative fever cut-off for tympanic temperature** in neonates, children and adults
  - as diagnostic **criterium for blood culture sampling** in neonates, children and adults for tympanic temperature

Note: The objective fever criterium is used in combination with other diagnostic criteria for blood culture sampling, see "TLM_PRO_044_Appendix 2_v4.0_Indications for blood culture sampling" and "TLM_PRO_044_Appendix 3_v3.0_Indications neonatal blood culture sampling".

The table below gives a short overview of the temperature cut-offs used as diagnostic criteria for blood culture sampling in neonates, children and adults per temperature measurement method.

**Fever & hypothermia cut-offs for neonates, children & adults per measurement method.**			
	**Rectal**	**Axillary**	**Tympanic**
**Fever**	> 38.0 °C (WHO)	> 37.5°C (WHO)	> 37.5°C
**Hypothermia**	< 36.0 °C (WHO)	≤ 35.5°C (WHO)	≤ 35.5°C

The rationale for these cut-offs as (one of the) diagnostic criteria for blood culture sampling, is based on the following :

- these temperature cut-offs correspond to the WHO definitions of fever and hypothermia for axillary and rectal temperature [2,6,7]
- alternative cut-offs for tympanic temperature increase diagnostic performance and are chosen in analogy with the alternative fever and hypothermia WHO cut-offs for axillary temperature
- most blood cultures are sampled upon arrival in referral hospitals, as a consequence:
  - many patients will have taken antipyretics before hospital arrival, which will lower their temperature upon arrival
  - most patients will have a history of fever in the past 48 hours, as such, these patients already respond to the fever criterium for blood culture sampling, irrespective of their temperature upon arrival
- erroneous tympanic measurement can reduce the measured tympanic temperature. Correct measurement is most difficult in children, while systemic and severe localized infections are also most difficult to recognize in children.

The appropriateness of these cut-offs will be evaluated during the prospective studies organized as part of the PhD of Bieke Tack in children under five admitted to Kisantu general referral hospital, DR Congo.

Product choice and selection of infrared tympanic thermometers [34]**:**

- Pediatric department of University Hospitals Leuven (UZ Leuven) opted for Genius 3 Tympanic Thermometer (Cardinal Health):

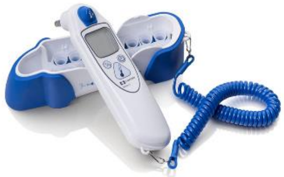

- Price: ± 300 euro ; Probe covers: ± 0.20 euro per piece
- Instructions for use and for cleaning
- Complies with ISO Standards
- Commercially available calibrator

- The ThermoScan Pro (Braun) is a good alternative. A more affordable alternative, assessed in tropical settings [14,24,32], is the ThermoScan 5 (Braun), however, this device is not intended for professional use and frequent disinfection and cannot be calibrated.

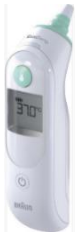

- Price: 60 euro ; Probe covers: : ± 0.20 euro per piece
- Instructions for use and cleaning
- Complies with ISO Standards
- Calibration services available
- Further investigations include:
  - ✓ possibilities of on-site calibration and internal quality control
  - ✓ standard operating procedure writing and training
  - ✓ ease-of-use testing

Conclusions:

1. We propose the use of infrared tympanic thermometers for clinical studies in low-resource settings.
2. We propose to use 37.5°C as an alternative fever cut-off and as a diagnostic criterium for blood culture sampling.
3. We propose to use the ThermoScan 5 (Braun) due to its affordability and existing data from tropical settings.
4. Further investigations should clarify possibilities of on-site calibration and internal quality control and ease-of-use. A standard operating procedure must be written and training must be provided.

Changes in comparison to last version:

- Addition of data from a recent meta-analysis on non-contact infrared thermometers [35]
- Addition of fever and hypothermia cut-offs as criterium for blood culture sampling

**Name and function**	**Date**	**Signature**
Author		
Bieke Tack (ITM ; pediatrics UZ Leuven)	10/07/2020<br>Last revision: 27/05/2021	
Reviewed by		
Jan Jacobs (ITM)	10/07/2020	
Approved by		
Karel Allegaert (pediatrics UZ Leuven)<br>Jaan Toelen (pediatrics UZ Leuven)		

References:

1 Ombelet S, Barbé B, Affolabi D, Ronat J-B, Lompo P, Lunguya O *et al.* Best Practices of Blood Cultures in Low- and Middle-Income Countries. *Front Med* 2019; **6**. doi:10.3389/fmed.2019.00131.

2 World Health Organization; *GLASS method for estimating attributable mortality of antimicrobial resistant bloodstream infections*. World health organization: Geneva, 2020.

3 Eddleston M, Davidson R, Brent A, Wilkinson R. *Oxford handbook of tropical medicine*. 3rd ed. Oxford University Press: Oxfors, 2011.

4 Simon C, Everitt H, van Dorp F. *Oxford handbook of general practice*. 3rd ed. Oxford University Press: Oxford, 2010.

5 Allegaert K, Casteels K, van Gorp I, Bogaert G. Tympanic, infrared skin, And temporal artery scan thermometers compared with rectal measurement in children: A real-life assessment. *Curr Ther Res - Clin Exp* 2014; **76**: 34–38.

6 World health organization. *Integrated Management of Childhood Illnesses*. First. World health organization: Geneva, 2014.

7 World health organization. *Pocket book of hospital care for children: guidelines for the management of common childhood illnesses*. 2nd ed. World health organization: Geneva, 2013.

8 Nimah MM, Bshesh K, Callahan JD, Jacobs BR. Infrared tympanic thermometry in comparison with other temperature measurement techniques in febrile children. *Pediatr Crit Care Med* 2006; **7**: 48–55.

9 El-Radhi AS, Barry W. Thermometry in paediatric practice. Arch. Dis. Child. 2006; **91**: 351–356.

10 Niven DJ, Gaudet JE, Laupland KB, Mrklas KJ, Roberts DJ, Stelfox HT. Accuracy of peripheral thermometers for estimating temperature: A systematic review and meta-analysis. Ann. Intern. Med. 2015; **163**: 768–777.

11 Hayward G, Verbakel JY, Ismail FA, Edwards G, Wang K, Fleming S *et al.* Non-contact infrared versus axillary and tympanic thermometers in children attending primary care: A mixed-methods study of accuracy and acceptability. *Br J Gen Pract* 2020; **70**: E236–E244.

12 National Institute of Clinical Excellence. Fever in under 5s: assessment and initial management. 2019https://www.nice.org.uk/guidance/ng143.

13 World health organization. *Emergency Global Supply Chain System*. World Health Organization: Geneva, 2020.

14 Abdulkadir MB, Johnson WBR. A comparative study of rectal tympanic and axillary thermometry in febrile children under 5 years of age in Nigeria. *Paediatr Int Child Health* 2013; **33**: 165–169.

15 Paes BF, Vermeulen K, Brohet RM, Van Der Ploeg T, De Winter JP. Accuracy of tympanic and infrared skin thermometers in children. *Arch Dis Child* 2010; **95**: 974–978.

16 Zhen C, Xia Z, Long L, Pu Y. Accuracy of infrared ear thermometry in children: a meta-analysis and systematic review. *Clin Pediatr (Phila)* 2014; **53**: 1158–65.

17 Craig J V., Lancaster GA, Taylor S, Williamson PR, Smyth RL. Infrared ear thermometry compared with rectal thermometry in children: A systematic review. *Lancet* 2002; **360**: 603–609.

18 Mogensen CB, Wittenhoff L, Fruerhøj G, Hansen S. Forehead or ear temperature measurement cannot replace rectal measurements, except for screening purposes. *BMC Pediatr* 2018; **18**: 15.

19 Bijur PE, Shah PD, Esses D. Temperature measurement in the adult emergency department: Oral, tympanic membrane and temporal artery temperatures versus rectal temperature. *Emerg Med J* 2016; **33**: 843–847.

20 Fortuna EL, Carney MM, Macy M, Stanley RM, Younger JG, Bradin SA. Accuracy of Non-contact Infrared Thermometry Versus Rectal Thermometry in Young Children Evaluated in the Emergency Department for Fever. *J Emerg Nurs* 2010; **36**: 101–104.

21 Geijer H, Udumyan R, Lohse G, Nilsagård Y. Temperature measurements with a temporal scanner: Systematic review and meta-analysis. *BMJ Open* 2016; **6**. doi:10.1136/bmjopen-2015-009509.

22 Hamilton PA, Marcos LS, Secic M. Performance of infrared ear and forehead thermometers: a comparative study in 205 febrile and afebrile children. *J Clin Nurs* 2013; **22**: 2509–2518.

23 Kiekkas P, Stefanopoulos N, Bakalis N, Kefaliakos A, Karanikolas M. Agreement of infrared temporal artery thermometry with other thermometry methods in adults: Systematic review. J. Clin. Nurs. 2016; **25**: 894–905.

24 Oyakhirome S, Profanter K, Kremsner PG. Short report: Assessment of fever in African children: Implication for malaria trials. *Am J Trop Med Hyg* 2010; **82**: 215–218.

25 Jefferies S, Weatherall M, Young P, Beasley R. A systematic review of the accuracy of peripheral thermometry in estimating core temperatures among febrile critically ill patients. *Crit Care Resusc* 2011; **13**: 194–199.

26 Shi D, Zhang LY, Li HX. Diagnostic test accuracy of new generation tympanic thermometry in children under different cutoffs: A systematic review and meta-analysis. *BMC Pediatr* 2020; **20**: 210.

27 Zhen C, Long L, Xia Z, Ya Jun Z, Jian S, Gui Ju C. Accuracy of infrared tympanic thermometry used in the diagnosis of fever in children: A systematic review and meta-analysis. *Clin Pediatr (Phila)* 2015; **54**: 114–126.

28 Teran CG, Torrez-Llanos J, Teran-Miranda TE, Balderrama C, Shah NS, Villarroel P. Clinical accuracy of a non-contact infrared skin thermometer in paediatric practice. *Child Care Health Dev* 2012; **38**: 471–476.

29 Chatproedprai S, Heamawatanachai K, Tempark T, Wananukul S. A Comparative Study of 3 Different Methods of Temperature Measurement in Children. *J Med Assoc Thai* 2016; **99**: 142–149.

30 Fletcher T, Whittam A, Simpson R, Machin G. Comparison of non-contact infrared skin thermometers. *J Med Eng Technol* 2018; **42**: 65–71.

31 Aw J. The non-contact handheld cutaneous infra-red thermometer for fever screening during the COVID-19 global emergency. J. Hosp. Infect. 2020; **104**: 451.

32 Chue AL, Moore RL, Cavey A, Ashley EA, Stepniewska K, Nosten F *et al.* Comparability of tympanic and oral mercury thermometers at high ambient temperatures. *BMC Res Notes* 2012; **5**. doi:10.1186/1756-0500-5-356.

33 Odinaka KK, Edelu BO, Nwolisa CE, Amamilo IB, Okolo SN. Temporal Artery Thermometry in Children Younger Than 5 Years. *Pediatr Emerg Care* 2014; **30**: 867–870.

34 World health organization. Hospital medical equipment - general information: thermometers, electronic, infrared. Geneva, 2012.

35 Aggarwal N , Garg M, Dwarakanathan V, Gautam N, Kumar S, Jadon RS, Gupta M, Ray Y, Diagnostic accuracy of non-contact infrared thermometers and thermal scanners: a systematic review and meta-analysis. Journal of Travel Medicine, 2020, 1–17.
