# Supplementary material for: Field Experiences with Handheld Diagnostic Devices to Triage Children under Five Presenting with Severe Febrile Illness in a District Hospital in DR Congo
Source: Diagnostics (Basel). 2022 Mar 18;12(3):746. doi: 10.3390/diagnostics12030746 (PMC8947034; doi:10.3390/diagnostics12030746)
Supplement: Supplementary file 1 [file diagnostics-12-00746-s001.zip › Supplement Proofs/220104_BT_Field experiences_S5.pdf]

INSTITUTE OF TROPICAL MEDICINE ANTWERP<br>INRB	**Titre:** Utiliser oxymètre Masimo Rad-G<br>**Premier auteur:** Bieke Tack<br>**Dernière révision:** 17/02/2021
	**Etude DeNTS:** Clinical decision support in non-typhoidal *Salmonella* bloodstream infections in children in sub-Saharan Africa: a prospective cohort study<br>**Etude TreNTS:** Treatment of non-typhoidal Salmonella bloodstream infections in children in sub-Saharan Africa: a prospective cohort study

1. Domaine et application

Ce document fournit les instructions pour mesurer la saturation en oxygène, la fréquence cardiaque et la fréquence respiratoire avec l'oxymètre de pouls Rad-G (Masimo).

La mesure de la saturation en oxygène du sang (SpO2) s'effectue selon le principe d'absorbance de la lumière pour déterminer le taux de saturation en oxygène. La source lumineuse émet la lumière dans deux longueurs d'onde, qui sont sélectivement absorbées par l'oxyhémoglobine et la désoxyhémoglobine. A l'aide du changement de son intensité optique après avoir passé le réseau des vaisseaux capillaires, la lumière du récepteur optique calcule le rapport entre l'oxyhémoglobine et l'hémoglobine totale.

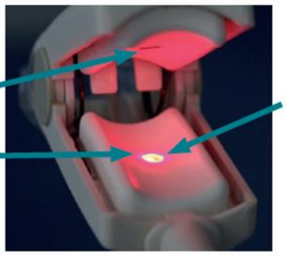

2. Responsabilité

Fonction	Activités
Infirmier(e) ou Médecin	• mesure la saturation oxygène, la fréquence cardiaque et la fréquence respiratoire<br>• enregistre les résultats dans le cahier des observations (« CRF »)<br>• averti le médecin traitant en cas d'hypoxie

3. Procédure

3.1 Matériel requis

Oxymètre Rad-G

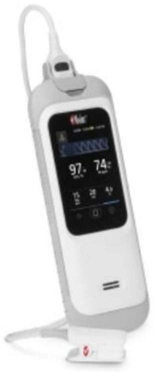

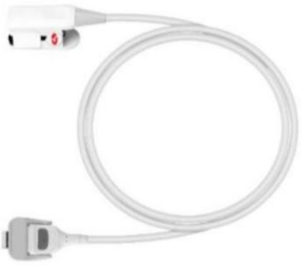

- Plage de mesure :
  - SpO2 : 0 -100%
  - Fréquence cardiaque : 25 - 240 /min
  - Fréquence respiratoire : 4 - 70 /min
- Stockage:
  - Température entre -20 et 140°C
  - Humidité entre 10 et 95 %
- Conditions de mesure:
  - Température ambiante entre 0 et 50°C et entre 0 et 40°C pendant chargement
  - Humidité entre 10 et 95%
- Alimentation:
  - Batterie Lithium : 24 heures
  - Comment recharger ?
    - Branchez le cordon d'alimentation CA dans le module d'entrée d'alimentation de l'oxymètre. Branchez aussi le cordon d'alimentation CA dans une source d'alimentation CA.
    - Temps pour charger complètement : 8 heures
- **Sonde Rad-G** : capteur de doigt à clip réutilisable. **Utilisez seulement cette sonde !**

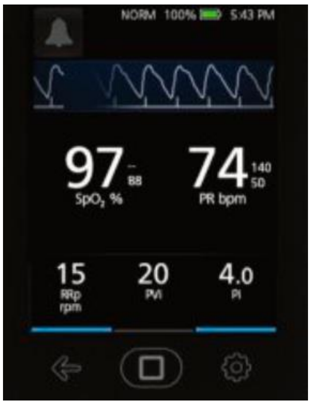

3.2 Préparation

1. **Allumez l’oxymètre** en appuyant sur la touche .
2. **Reliez la sonde à l’oxymètre.**
3. Assurez-vous que l’oxymètre est dans le mode correcte : « APOD »
   Si elle ne se trouve pas dans la mode correcte :
   - ➢ Appuyez sur
   - ➢ Selectionez « Additional settings »
   - ➢ Selectionez APOD comme “Sensitivity mode” et confirmer ce choix en cliquant sur « OK »
   - ➢ Retournez à l’écran principal en appuyant sur

3.3 Procédure

1. **Attachez la sonde au patient:**
   - Choisissez un site bien irrigué et qui ne risque pas d’entraver les mouvements de l’enfant
   - Veillez à choisir un site couvrant complètement la fenêtre du détecteur de la sonde
   - Vérifiez que le site est propre
   - Site de préférence par plage de poids / âge :
     - ➢Nourrisson : 3 – 10 kg : gros orteil, sinon : pouce
     - ➢Enfant : 10 – 50 kg : doigt de la main non-dominante, sinon : autre doigt
   - Faites attention que la côté avec indication de l’ongle est placée au côté de l’ongle

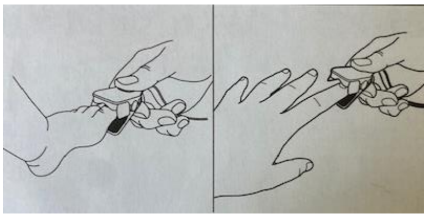

2. **Attendez jusqu’à ce que vous voyez :**
   - **une belle courbe régulière**
   - **avec un bon signal :** bonne hauteur des barres verticales de l’indicateur de qualité de signal

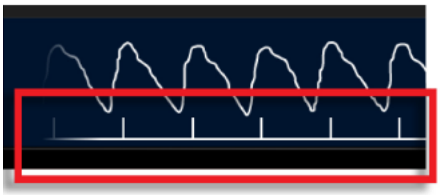

Belle courbe régulière et bon qualité de signal (marqué par le cadre rouge)

   Si la **courbe est irrégulière et/ou le signal n’est pas bien détecté**, la saturation en oxygène (SpO2), la fréquence cardiaque (PR) et la fréquence respiratoire (FR) ne sont pas fiable !

   Voici les causes principales pour **des courbes irrégulières** et des actions pour résoudre le problème :

   - L’enfant bouge, tremble, pleure, … :
     - ➢ Attendez et laissez l’enfant le temps pour se calmer ou d’être calmé par son parent / soignant (en allaitant l’enfant, parlant avec l’enfant, chantant une chanson pour l’enfant, …)
     - ➢ Tenez lui la main / le pied ou demandez au parent / soignant de lui tenir la main / le pied.
   - La sonde est mal positionnée :
     - ➢ Repositionnez-la, si nécessaire sur une autre place, par exemple un autre doigt ou l’orteil
   - Evitez les lumières trop fortes en direction de la sonde, car cela peut interférer avec le détecteur de lumière de la sonde.

   Un **signal faible** peut être lié à une mauvaise circulation, par exemple quand l’enfant souffre d’hypovolémie, hypothermie ou anémie sévère. Essayez de :
   - Repositionner la sonde sur une site mieux circulée
   - Laissez la sonde bien positionnée sans plus y toucher pendant une minute.

3. **Lisez la saturation en oxygène (SpO2), la fréquence cardiaque (PR) et la fréquence respiratoire (RRp)** quand vous avez une courbe régulière et un bon signal et les chiffres sont affichés en blanc.

Les paramètres sont affichés en bleu si il s'agit d'une estimation préliminaire et en blanc si il s'agit d'une valeur définitive. Les paramètres sont affichés en grand. Les petits chiffres à côté de la saturation et fréquence cardiaque indiquent les limites d'alarme.

4. **Enregistrez les données** dans le registre des signes vitaux et dans le cahier d'observations (« CRF ») de l'enfant. Enregistrez aussi les éventuelles difficultés que vous avez rencontré pendant que vous avez prélevé les paramètres.
5. **Eteignez l'oxymètre** en appuyant sur la touche .

Si vous n'avez pas réussi à obtenir une belle courbe avec un bon signal malgré plusieurs essais, ou si vous n'obtenez pas la fréquence respiratoire (valeur définitive) malgré une belle courbe avec un bon signal :

- Enregistrez dans le cahier d'observations de l'enfant (« CRF ») que vous n'avez pas réussi à mesurer le(s) paramètre(s) et la raison pour laquelle vous n'avez pas réussi.
- La fréquence cardiaque peut être compté manuellement (auscultation du cœur, palpation du pouls au niveau du poignet / artère brachiale). Comptez la fréquence cardiaque pendant 30 secondes et multipliez ce chiffre par deux pour obtenir la fréquence cardiaque par minute. Enregistrez cette fréquence cardiaque dans le cahier d'observations de l'enfant (« CRF ») et mentionnez qu'il s'agit d'une mesure manuelle.
- La fréquence respiratoire peut être compté manuellement. Comptez les mouvements thoracique (si nécessaire comptez les inspirations/expirations en auscultant la respiration) pendant 30 secondes et multipliez ce chiffre par deux pour obtenir la fréquence respiratoire par minute. Enregistrez cette fréquence respiratoire dans le cahier d'observations de l'enfant (« CRF ») et mentionnez qu'il s'agit d'une mesure manuelle.

Remarque importante : Nous ne connaissons pas encore la fiabilité de la mesure de la fréquence respiratoire. Pour comparer la mesure automatique avec Rad-G et la fréquence respiratoire compté par la méthode manuelle, comptez aussi la fréquence respiratoire manuellement pour chaque enfant et enregistrez ceci dans le registre des signes vitaux et dans le CRF.

Interprétation de la saturation en oxygène :
Hypoxie : Avertissez le clinicien traitant immédiatement. Normalement, l'oxymètre déclenche une alarme.

- Hypoxie = saturation en oxygène ≤ 90%

Interprétation de la fréquence cardiaque:
Tachycardie : Avertissez le clinicien traitant immédiatement si accompagnée d'un temps de recoloration > 3 secondes et peau froide.

- Enfant < 12 mois: tachycardie = fréquence cardiaque > 160/min.
  Normalement, l'oxymètre déclenche une alarme.
- Enfant ≥ 12 mois – 5 ans : tachycardie = fréquence cardiaque > 120/min.
  Attention : normalement l'oxymètre ne déclenchera pas d'alarme (limite supérieure = 160/min).

Interprétation de la fréquence respiratoire:
Tachypnée : Avertissez le clinicien traitant immédiatement si accompagnée de détresse respiratoire sévère ou hypoxie.

- Enfant < 2 mois: tachypnée = fréquence respiratoire ≥ 60/min.
  Normalement, l'oxymètre déclenche une alarme si la fréquence respiratoire est ≥ 40/min.
- Enfant ≥2 mois et < 12 mois: tachypnée = fréquence respiratoire ≥ 50/min.
  Normalement, l'oxymètre déclenche une alarme si la fréquence respiratoire est ≥ 40/min.

- Enfant ≥ 12 mois – 5 ans : tachypnée = fréquence respiratoire ≥ 40/min.
  Normalement, l'oxymètre déclenche une alarme si la fréquence respiratoire est ≥ 40/min.

4. Alarmes

L'alarme permet de donner une indication visuelle et auditive au personnel médical lorsque les signes vitaux du patient semblent anormaux ou lorsque l'oxymètre présente un problème technique.

1. **Alarme physiologique:**
   La saturation en oxygène, la fréquence cardiaque ou la fréquence respiratoire en dehors des limites prédéfinies :
   - Saturation en oxygène: alarme si SpO2 < 90%
   - Fréquence cardiaque: alarme si fréquence cardiaque (PR) > 160 /minute ou < 60/minute
   - Fréquence respiratoire: alarme si fréquence respiratoire (RR) ≥ 40 /minute ou < 6/minute
2. **Alarme technique:**
   Alarme si la probe n'est plus attaché au patient, un alarme « Sensor off patient » apparaît

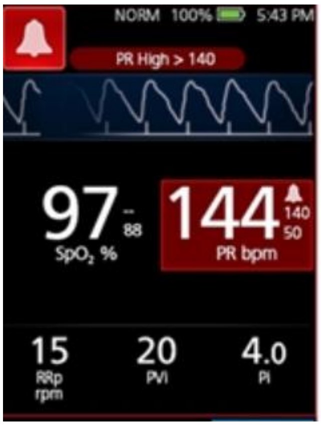

Indications visuelles d'alarme:

- Un message d'alarme apparaît sur l'écran
- Le paramètre qui provoque l'alarme clignote et est marqué en rouge

Indications auditives d'alarme :

- Emission d'un son

Vous pouvez éteindre l'alarme temporellement :

- Appuyez sur la touche 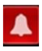 sur l'écran :
  L'alarme est temporellement suspendu pour 120 secondes :
  - L'alarme sonore est suspendue, mais les alarmes visuelle et textuelle seront toujours effectives.
  - L'icône 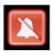 est affiché ensemble avec le temps de pause restant (en secondes) dans le coin supérieur gauche de l'écran
- Appuyez de nouveau sur la touche 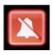 pour rallumer l'alarme avant que les 120s soient passées.

5. Entretien

Chaque jour, vous devez nettoyer/désinfecter l'appareil et la sonde:
Assurez-vous d'abord que l'oxymètre est éteint et qu'aucun accessoire ne soit relié à l'appareil.

Nettoyage et désinfection de l'oxymètre et la sonde :

- Nettoyez délicatement à l'aide d'une lingette imbibée d'alcool isopropylique 70% ou éthanol 70%
- Assurez-vous que la lingette n'est pas trop humide et évitez à tout prix que la prise ou le point de connexion du câble de la sonde soit mouillé.
- Ne jamais immerger l'oxymètre et la sonde dans un liquide. Ne jamais verser de liquide sur l'oxymètre ou la sonde.

6. Références

- Mode d'emploi de l'oxymètre Rad-G (Masimo)
- World health organization. Pocket book of hospital care for children: guidelines for the management of common childhood illnesses. 2nd ed. World health organization: Geneva, 2013.

7. Histoire du document

**Name and function**	**Date**	**Comments**
Auteur original		
Bieke Tack	17/02/2021	
Révisé par		
Idzi Potters	18/02/2021	
Approuvé par		
Jan Jacobs		
