# Supplementary material for: Field Experiences with Handheld Diagnostic Devices to Triage Children under Five Presenting with Severe Febrile Illness in a District Hospital in DR Congo
Source: Diagnostics (Basel). 2022 Mar 18;12(3):746. doi: 10.3390/diagnostics12030746 (PMC8947034; doi:10.3390/diagnostics12030746)
Supplement: Supplementary file 1 [file diagnostics-12-00746-s001.zip › Supplement Proofs/220104_BT_Field experiences_S6.pdf]

INSTITUTE OF TROPICAL MEDICINE ANTWERP<br>INRB	**Titre:** Mesurer la glycémie avec le glucomètre Accu-chek Performa<br>**Premier auteur:** Bieke Tack<br>**Dernière révision:** 17/02/2021
	**Etude DeNTS:** Clinical decision support in non-typhoidal *Salmonella* bloodstream infections in children in sub-Saharan Africa: a prospective cohort study<br>**Etude TreNTS:** Treatment of non-typhoidal Salmonella bloodstream infections in children in sub-Saharan Africa: a prospective cohort study

1. Domaine et application

Ce document fournit les instructions pour mesurer la glycémie avec le glucomètre Accu-chek Performa.

2. Responsabilité

Fonction	Activités
Infirmier / Infirmière	• fait la prise de sang capillaire<br>• suit la procédure pour mesurer la glycémie avec le glucomètre Accu-chek Performa.<br>• enregistre les résultats dans le cahier des observations (« CRF »)<br>• informe le clinicien traitant en cas d'hypoglycémie

3. Procédure

3.1 Précautions

Tous les échantillons de sang sont potentiellement infectieux. Mettez des gants pendant toute la procédure et lavez-vous les mains avec eau et savon ou désinfectez-vous les mains avec un désinfectant approprié avant et après la procédure.

3.2 Préparation

3.2.1 Matériel fourni et conservation

1. Le lecteur Accu-Chek Performa:
   Stockage : température entre -25 et 70°C
   Conditions de mesure : température entre 8 et 44°C, humidité entre 10 et 90%, valeur hématocrite du patient entre 10 et 65%
   Alimentation : Pile au lithium de 3 volts (type pile bouton CR2032), regardez dans le mode d'emploi pour voir comment remplacer la pile.

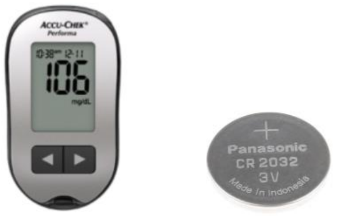

2. Tube de bandelettes réactives:
   Utilisez uniquement des bandelettes réactives Accu-Chek Performa .
   Stockage : température entre 2 et 32°C, ne pas réfrigérer ou congeler !
   Durabilité : les bandelettes restent utilisables jusqu'à la date de péremption imprimée sur le flacon, même après ouverture. Néanmoins, évitez de laisser le tube ouvert ; refermez-le hermétiquement et aussi vite que possible après chaque usage.

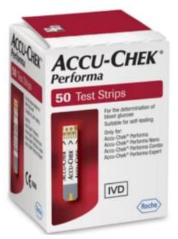

3.2.2 Matériel supplémentaire requis

1. Gants non-stériles, à usage unique, lancettes stériles, récipient pour objets tranchants, compresse alcoolisée (70% ethanol ou 70% alcool isopropylique) et coton sec
2. Accu-Chek Performa solutions de contrôle 1 et 2
   Utilisez uniquement la solution de contrôle Accu-Chek Performa.
   Stockage : température entre 2 et 32°C, ne pas congeler !
   Durabilité : 3 mois après ouverture du flacon. Refermez toujours après utilisation.

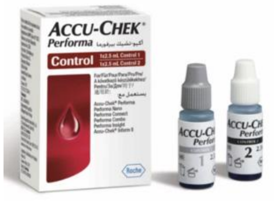

3.3 Procédure

Regardez aussi le vidéo d'entrainement fait par la firme : https://www.youtube.com/watch?v=N-IEe5tb15k

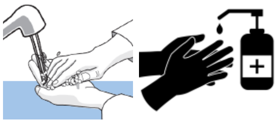	Lavez-vous les mains avec eau et savon ou désinfectez-vous les mains avec un désinfectant approprié avant de commencer.
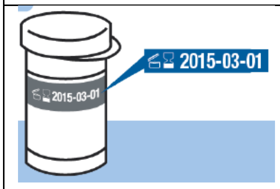	Vérifiez la date de péremption des bandelettes. Si la date de péremption est dépassée, jetez le tube avec les bandelettes périmées et utilisez une bandelette d'un tube non-périmé.
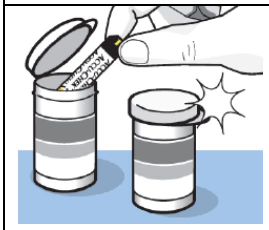	Retirez une bandelette réactive du tube de bandelettes.<br><br>Refermez le tube hermétiquement.<br><br>Utilisez la bandelette réactive aussitôt après l'avoir retirée du tube.
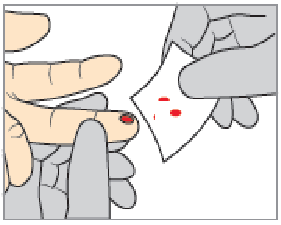	Faites la prise de sang capillaire comme décrit dans la procédure concernée.<br><br>Pour éviter l'interférence des aliments et alcool sur le doigt avec la glycémie, nettoyez d'abord le doigt/talon 2 fois avec une compresse préimbibée d'alcool isopropylique 70% et désinfectez-le finalement avec une 3e compresse préimbibée d'alcool isopropylique 70%. Faites bien attention d'attendre jusqu' à ce que le doigt soit complètement sec (tout l'alcool évaporisé).<br><br>Faites les autres tests comme décrit dans les procédures concernées :<br>– Test diagnostique rapide de paludisme<br>– Détermination d'hémoglobine avec HemoCue Hb 801
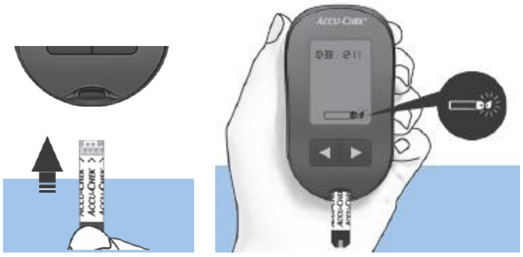	Sans avoir appliqué du sang, ni du solution de contrôle, insérez la bandelette réactive dans le lecteur dans le sens des flèches. Le lecteur s'allume et émet un signal sonore.<br><br>Le symbole d'une goutte de sang clignotante s'affiche.
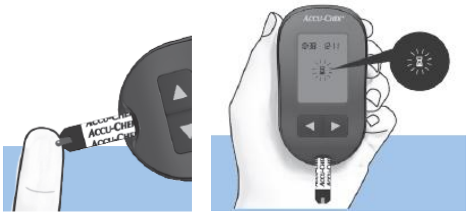<br>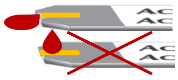 **Correcte**<br>**Pas correcte !**	Mettez le bord de la fenêtre jaune de la bandelette en contact avec la goutte de sang obtenue. Ne déposez pas de sang au-dessus de la bandelette !<br><br>Le lecteur émet un signal sonore et le symbole ⌛ clignote dès qu'une quantité suffisante de sang a été aspirée par la bandelette réactive.

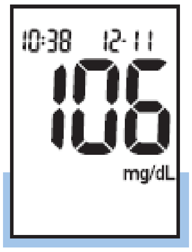	Le résultat s'affiche à l'écran au bout de 5 secondes. Enregistrez le résultat (en mg/dl) dans le cahier d'observations (« CRF ») du patient.<br><br>Retirez et éliminez la bandelette usagée. Le lecteur s'éteint automatiquement 5 secondes après le retrait de la bandelette.<br><br>Informez le clinicien traitant si la glycémie est < 50 mg/dl.

Remarque:

- Les résultats glycémiques sont enregistrés du plus récent au plus ancien, vous devez donc régler l'heure et la date du lecteur correctement. Regardez dans le mode d'emploi comment régler la date et l'heure.
- Pour consulter les résultats enregistrés, il faut :
    1. Allumer le lecteur : appuyez brièvement sur la touche « marche/arrêt » dans le coin droit supérieur du lecteur.
    Si vous y appuyez trop longtemps, vous accéder au mode réglage. Vous pouvez quitter du mode réglage en appuyant de nouveau sur la touche (maintenez-la enfoncée jusqu'à ce que le symbole de la bandelette réactive clignotante s'affiche à l'écran).
    2. Appuyez brièvement sur la touche . Le résultat le plus récent s'affiche à l'écran.
    3. Continuez à appuyer brièvement sur la touche pour afficher les résultats antérieurs un à un du plus récent au plus ancien.

4. Entretien

- Protégez le lecteur contre la poussière. Gardez-le alors toujours dans sa poche.
- Chaque jour, vous devez nettoyer l'extérieur du lecteur délicatement à l'aide d'un chiffon doux légèrement imbibé d'éthanol 70% ou alcool isopropylique 70%. Enlevez tout excès de liquide. Faites un contrôle de qualité après avoir nettoyé le lecteur.
- Ne jamais :
    - faire pénétrer le moindre de liquide dans n'importe quel ouverture du lecteur
    - vaporiser une solution nettoyante directement sur le lecteur
    - immerger le lecteur dans un liquide

5. Contrôle de qualité

Quand faire un test de contrôle ?

Faites un test de contrôle pour vérifier si le lecteur et les bandelettes fonctionnent correctement chaque fois que :

- Vous avez nettoyé le lecteur.
- Vous ouvrez un nouveau tube de bandelettes réactives.
- Vous avez laissé le tube de bandelettes réactives ouvert.
- Vous pensez que les bandelettes réactives sont endommagées.
- Vous souhaitez vérifier le fonctionnement du lecteur et des bandelettes réactives, parce que vous avez l'impression que les résultats ne sont pas corrects.
- Vos bandelettes réactives ont été exposées à des températures excessives ou à l'humidité.
- Vous avez laissé tomber le lecteur.

Procédure du test de contrôle :

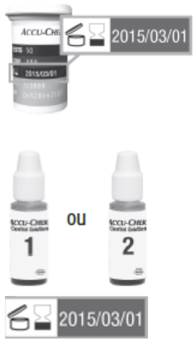	Vérifiez la date de péremption sur le tube de bandelettes réactives. Si nécessaire, jetez le tube avec les bandelettes périmées et utilisez une bandelette d'un tube non-périmé.<br>Vérifiez aussi la date de péremption de la solution de contrôle. Jetez la solution et ouvrez un nouveau flacon si :<br>- Plus de 3 mois sont dépassés après l'ouverture du flacon<br>ou<br>- La date de péremption est dépassée
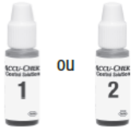 **/02/2021**	Faites d'abord le test de contrôle avec lasolution de contrôle 1, puis répétez la procédure avec la solution de contrôle 2.<br>Si vous allez ouvrir un nouveau flacon de solution de contrôle, écrivez la date d'ouverture sur le flacon.
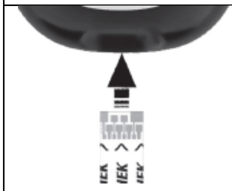	Insérez la bandelette réactive dans le lecteur en respectant le sens des flèches.
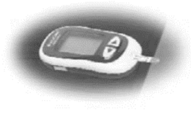	Mettez le lecteur sur une surface stable et horizontale, comme une table.
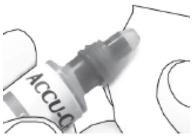	Enlevez le bouchon du flacon de solution de contrôle.<br>Essuyez l'embout du flacon avec un mouchoir en papier.<br>Pressez le flacon doucement pour faire sortir une petite goutte qui se forme à l'embout du flacon.
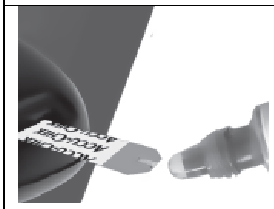	Mettez la goutte de sang obtenue en contact avec le bord de la fenêtre jaune de la bandelette. Ne déposez pas de sang au-dessus de la bandelette!<br>Le lecteur émet un signal sonore et le symbole ⌛ clignote dès qu'une quantité suffisante de liquide de contrôle a été aspirée par la bandelette réactive.
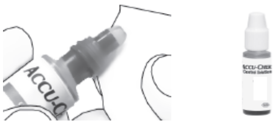	Essuyez l'embout du flacon avec un mouchoir en papier. Refermez bien le flacon.
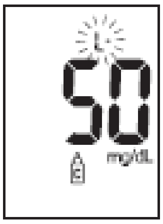	Le résultat de contrôle s'affiche à l'écran en même temps que le symbole du flacon de solution de contrôle et la lettre L clignotante. *(Le lecteur fait automatiquement la différence entre la solution de contrôle et le sang.)*<br>Ne retirez pas encore la bandelette réactive.<br>Appuyez une fois sur la touche ▶ pour indiquer qu'il s'agit d'un résultat de contrôle de niveau 1. Appuyez deux fois sur la touche ▶ pour indiquer qu'il s'agit d'un résultat de contrôle de niveau 2.

<table>
<tr>
<td>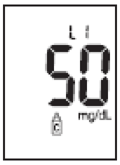 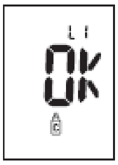</td>
<td>
Appuyez brièvement sur la touche ⓘ (au coin supérieur droit du lecteur) pour confirmer le niveau dans le lecteur.

L’indication « OK » et le résultat de contrôle s’affichent à l’écran en alternance si le résultat de contrôle se situe dans l’intervalle acceptable.
L’indication « Err » et le résultat de contrôle s’affichent à l’écran en alternance si le résultat de contrôle se situe hors de l’intervalle acceptable. Consultez le mode d’emploi pour détecter l’origine possible de l’erreur. En cas de problèmes que vous ne pouvez pas immédiatement résoudre, contactez l’investigateur principal, ou en cas d’absence, le superviseur local ou le superviseur de site.

Retirez et éliminez la bandelette usagée. Le lecteur s’éteint automatiquement 5 secondes après le retrait de la bandelette.
</td>
</tr>
</table>

Messages d’erreurs

Écran	Résolution du problème
Le lecteur ne s’allume pas ou l’écran reste vide.	• La pile est à plat. Insérez une nouvelle pile.<br>• L’écran est défectueux. Contactez Roche.<br>• Le lecteur est défectueux. Contactez Roche.<br>• Températures excessives. Déplacez le lecteur dans un endroit plus tempéré.
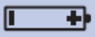	La pile est presque à plat. Remplacez la pile rapidement.
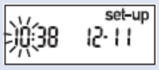	Le lecteur est en mode réglage, en attente de modification ou de confirmation de réglages.
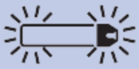	Le lecteur est prêt pour l’insertion d’une bandelette réactive.
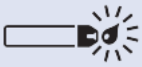	Le lecteur est prêt à aspirer une goutte de sang ou de solution de contrôle.
HI	La glycémie peut être supérieure à l’intervalle de mesure du système. Reportez-vous au chapitre 2, Résultats glycémiques inhabituels.
LO	La glycémie peut être inférieure à l’intervalle de mesure du système. Reportez-vous au chapitre 2, Résultats glycémiques inhabituels.
ⓘ	La glycémie est inférieure au seuil d’hypoglycémie (glycémie basse) qui a été défini.<br>Informez le clinicien traitant si la glycémie est < 50
*	Ce résultat glycémique a été marqué de manière générale.

Le lecteur procède automatiquement à un autodiagnostic à chaque mise sous tension et vous avertit de tout problème :

- **E-1 :** La bandelette réactive est peut-être endommagée ou mal insérée. Retirez la bandelette réactive et réinsérez-la ou remplacez-la si elle est endommagée.
- **E-3 :** Il est possible que la glycémie soit extrêmement élevée ou qu’une erreur soit survenue au niveau du lecteur ou de la bandelette réactive. Recommencez la mesure de glycémie. Si le résultat de la deuxième mesure de glycémie ne correspond pas à l’état de santé du patient, effectuez un test de contrôle avec la solution de contrôle et une nouvelle bandelette réactive. Si le résultat de contrôle se situe dans l’intervalle acceptable, assurez-vous que vous avez bien suivi les différentes étapes de réalisation de la mesure de glycémie et recommencez la mesure de glycémie avec une nouvelle

bandelette réactive. Si le message E-3 s'affiche de nouveau après votre mesure de glycémie, informez le clinicien traitant du patient que le patient souffre probablement d'hyperglycémie.

- **E-4 :** La bandelette réactive n'a pas aspiré suffisamment de sang ou de solution de contrôle pour que la mesure de glycémie ou le test de contrôle soit possible. Ou bien l'aspiration suffisante de sang ou de solution de contrôle s'est produite après le démarrage de la mesure de glycémie ou du test de contrôle. Éliminez la bandelette réactive et recommencez la mesure de glycémie ou le test de contrôle.
- **E-6 :** L'aspiration du sang ou de la solution de contrôle par la bandelette réactive a été effectuée avant que ne s'affiche le symbole de la goutte clignotante à l'écran. Éliminez la bandelette réactive et recommencez la mesure de glycémie ou le test de contrôle.
- **E-7 :** Une défaillance électronique s'est produite ou, ce qui est rare, une bandelette réactive usagée a été retirée du lecteur et réinsérée. Éteignez le lecteur, puis rallumez-le ou retirez la pile pendant 20 secondes et réinstallez-la. Effectuez une mesure de glycémie ou un test de contrôle.
- **E-8** : La température est supérieure ou inférieure aux températures préconisées pour l'utilisation du système. Déplacez-vous dans un endroit où la température est conforme à l'intervalle de températures préconisé, attendez 5 minutes et recommencez la mesure de glycémie ou le test de contrôle. N'essayez pas d'accélérer le réchauffement ou le refroidissement du lecteur.
- **E-9 :** La pile est presque à plat. Remplacez la pile maintenant. Si le message s'affiche de nouveau après le remplacement de la pile, retirez à nouveau la pile, appuyez sur n'importe quelle touche du lecteur, puis réinstallez la pile.
- **E-10 :** Il est possible que les réglages de l'heure et de la date soient erronés. Assurez-vous que l'heure et la date sont correctes et corrigez si nécessaire.

6. Références

- Mode d'emploi et guide de démarrage Accu-Chek Performa

7. Histoire du document

- Version 2.0 :

Partie sur nettoyage et désinfection ajoutée parce que mauvaise nettoyage et désinfection peuvent fausser les résultats.

Observation pendant premières 2 semaines de l'étude DeNTS : valeurs élevés : > 200 mg/dl chez enfants qui ne sont pas gravement malade :

- Alcool de désinfection peut baisser la glycémie (Foos et al 2017)
- Aliments sur le doigt (de l'enfant ou l'infirmier/médecin qui prélève) peuvent fortement augmenter la glycémie mesurée.

**Name and function**	**Date**	**Comments**
Auteur original		
Bieke Tack<br>Version 2.0	25/11/2020<br>17/02/2021	
Révisé par		
Idzi Potters	11/12/2020	
Approuvé par		
Jan Jacobs		
