# Supplementary material for: Field Experiences with Handheld Diagnostic Devices to Triage Children under Five Presenting with Severe Febrile Illness in a District Hospital in DR Congo
Source: Diagnostics (Basel). 2022 Mar 18;12(3):746. doi: 10.3390/diagnostics12030746 (PMC8947034; doi:10.3390/diagnostics12030746)
Supplement: Supplementary file 1 [file diagnostics-12-00746-s001.zip › Supplement Proofs/220104_BT_Field experiences_S7.pdf]

	Titre: Détermination de l'hémoglobine avec HemoCue Hb 801<br>Premier auteur: Bieke Tack<br>Dernière révision: 17/02/2021
	**Etude DeNTS:** Clinical decision support in non-typhoidal Salmonella bloodstream infections in children in sub-Saharan Africa: a prospective cohort study<br>**Etude TreNTS:** Treatment of non-typhoidal Salmonella bloodstream infections in children in sub-Saharan Africa: a prospective cohort study

1. Domaine et application

Ce document fournit les instructions pour assurer la sécurité et la précision de la mesure de l'hémoglobine en utilisant le système HemoCue Hb801. Dans cet appareil, l'absorbance du sang total est mesurée au point isobestique Hb/HbO_2. L'absorbance, corrigée pour la turbidité, est proportionnelle à la quantité d'hémoglobine présente dans le sang .

2. Responsabilités

Fonction	Activités
Infirmier / infirmière	• fait la prise de sang capillaire<br>• suit la procédure du système HemoCue Hb 801<br>• enregistre les résultats dans le cahier des observations (« CRF »)<br>• suit les règles d'assurance de qualité et tient un registre de tous les contrôles de qualité<br>• suit les procédures d'entretien

3. Procédures

3.1 Précautions

Tous les échantillons de sang sont potentiellement infectieux.
Mettez des gants pendant toute la procédure et lavez-vous les mains avec de l'eau et du savon ou désinfectez-vous les mains avec un désinfectant approprié avant et après la procédure.

3.2 Matériel et échantillons

3.2.1 Matériel fourni et conservation

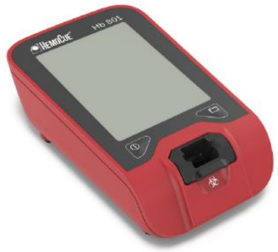

<u>L'appareil HemoCue Hb 801</u>:
Stockage : Entre 0 – 50 °C. Humidité : jusqu'à 90% à 25°C, jusqu'à 75% à 40°C.
Fonctionnement : Entre 10 – 40 °C. Humidité : jusqu'à 90% à 25°C, jusqu'à 75% à 40°C.
Alimentation : Cable USB et adaptateur secteur / ordinateur ou 3 piles AA

<u>Microcuvettes HemoCue Hb 801</u>:
Stockage :

- À température ambiante (15 - 40°C). Ne pas mettre au réfrigérateur.
- Utilisez avant la date de péremption.
  Après ouverture du flacon, les microcuvettes restent stables pendant 3 mois.
  Notez la date d'ouverture sur le flacon. Ne laissez jamais le flacon ouvert ; refermez toujours le flacon.

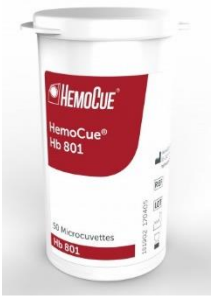

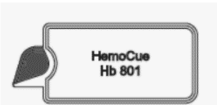

4 x 50 microcuvettes par boite

3.2.2 Matériel supplémentaire requis

- Gants non-stériles, à usage unique
- Lancettes stériles
- Récipient pour objets tranchants
- Compresse alcoolisée (ethanol 70% ou alcool isopropylique 70%)

- Coton sec
- Solutions de contrôle : Eurotrol Hb 801 control Low / Normal

3.3 Procédure

Vérifiez la date de péremption des microcuvettes et la date d'ouverture du flacon. Jetez les microcuvettes quand elles sont périmées ou quand la date d'ouverture a dépassé les trois mois. Dans ce cas-là, prenez un nouveau lot de microcuvettes non-périmé pour faire les mesures.

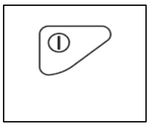 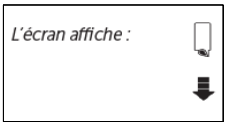 ***Touche "marche/arret"*** ***Mode "Prêt"***	Placez l'appareil HemoCue sur une surface plane, horizontale et stable.<br><br>Allumez l'l'appareil : appuyez sur la touche marche/arrêt jusqu'à ce que tous les segments de l'écrans s'affichent, puis relâchez et attendez quelques secondes que l'appareil soit en mode « Prêt ».
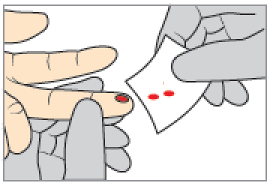	Faites la prise de sang capillaire comme décrit dans la procédure concernée. Après la piqure, essuyez la première goutte de sang.<br><br>Note :<br>Collectez d'abord le sang pour l'Hemocue, puis pour le test de diagnostic rapide de paludisme, et finalement pour le contrôle de glycémie.<br>Ne massez et ne serrez pas trop le doigt de l'enfant pour éviter l'extravasation de liquide extracellulaire ou hémoconcentration.
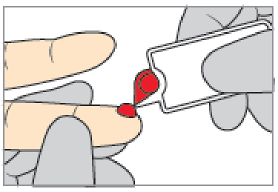	Remplissez la microcuvette.<br><br>Assurez-vous que la goutte de sang est suffisamment grosse pour remplir entièrement la microcuvette en une fois.
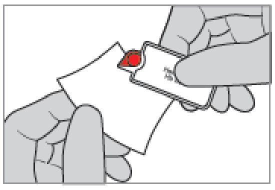	Essuyez le surplus de sang à l'extérieur de la microcuvette.<br><br>Veillez à ne pas aspirer de sang contenu dans la microcuvette.
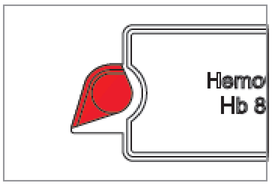	Faites une inspection visuelle :<br>– Si la microcuvette n'est pas entièrement rempli, ne la remplissez pas une deuxième fois, mais jetez la et remplissez une nouvelle microcuvette.<br>– Si la microcuvette contient des bulles d'air, jetez la et remplissez une nouvelle microcuvette.
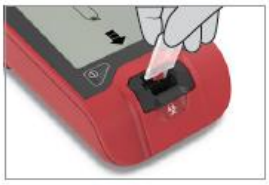	Assurez-vous que l'appareil est en mode « Prêt ».<br><br>Insérez la microcuvette remplie dans le support des microcuvettes et appuyez. Le résultat s'affiche en une seconde.<br><br>*Attention : La microcuvette doit être insérée au maximum 40 secondes après avoir rempli la microcuvette.*
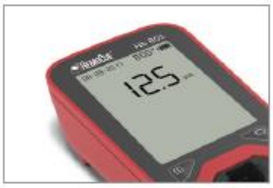	Lorsqu'un résultat s'affiche, retirez et jetez la microcuvette. Le résultat reste affiché pendant 10 secondes après la mesure.

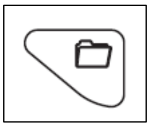 *Touche "Résultats précédents"*	Enregistrez le résultat (en g/dl) après avoir effectué le test de glycémie et le test de diagnostic rapide pour le paludisme. L'appareil HemoCue garde le résultat dans sa mémoire.<br><br>Vous pouvez consultez le résultat par après, en appuyant la touche « Résultats précédents ». Le numéro de séquence « 0001 » est toujours attribué au résultat le plus récent. Pour quitter les résultats précédents, appuyer simultanément sur les deux touches « Résultats précédents » et « Marche/Arrêt ».<br><br>Informez le clinicien traitant si l'hémoglobine est ≤ 5 g/dl.

3.4 Entretien

L'appareil HemoCue doit être nettoyé et désinfecté chaque jour.
Nettoyage/Désinfection :

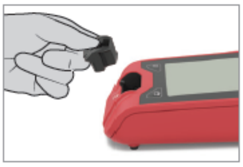	Mettez l'appareil hors tension .<br><br>Retirez le support de microcuvette.
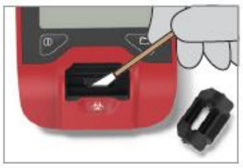	Prenez un écouvillon et imbibez-le légèrement d'éthanol 70% ou alcool isopropylique 70%.<br><br>Nettoyez toutes les surfaces dans la cavité : veillez à nettoyer jusqu'au fond.
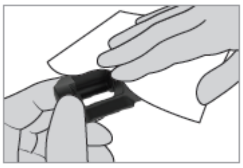<br>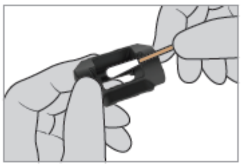	Essuyez le support de microcuvette avec éthanol ou isopropyl alcohol 70%.<br><br>Nettoyez le support de microcuvette avec éthanol 70% ou alcool isopropylique 70%.<br><br>Laisser le support de microcuvette sécher hors de l'appareil. Remettez-le quand tout a seché.
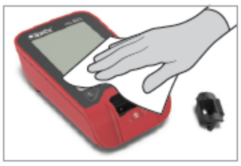	Nettoyez/Désinfectez toutes les surfaces extérieures de l'appareil HemoCue avec un chiffon imbibé légèrement avec de l'éthanol 70% ou avec de l'alcool isopropylique 70%.
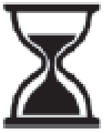	Attendez jusqu'à toutes les parties de l'analyseur sont sont sèches.<br><br>Remettez le support de microcuvette en place.<br><br>Mettez l'analyseur sous tension.

3.5 Contrôle de qualité

Chaque jour, après le nettoyage et désinfection, il faut faire un contrôle de qualité avec les solutions de contrôle recommandées: Hemotrol WB 801 control Low et Normal.

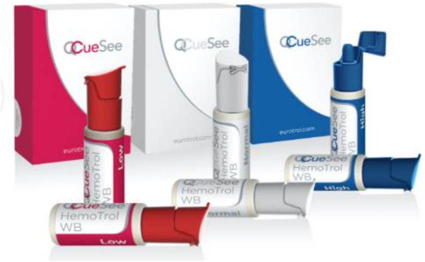

Attention : Pour des raisons de prévention d'infection et des performances optimales, Hemotrol doit être traité avec les mêmes mesures d'hygiène et la même ponctualité d'un échantillon provenant d'un patient.

Conservation:
- Stockage des flacons fermés entre 2 – 8 °C, voir la date de péremption indiquée sur l'étiquette du flacon.
- Après ouverture : stable pendant 30 jours à une température comprise entre 2 et 8°C

Procédure :

1. Équilibrez le flacon pendant 15 minutes pour le laisser atteindre une température de 15 à 30°C.
2. Immédiatement avant utilisation, agitez manuellement le flacon pendant au moins 30 secondes afin de bien en homogénéiser le contenu.
3. Maintenez le flacon verticale et tapotez le capuchon pour éliminer le liquide de l'embout du flacon compte-gouttes.
4. Ouvrez délicatement le capuchon sans presser le flacon.
5. Éliminez la première goutte du liquide de contrôle de qualité.

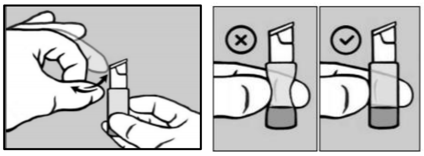

6. Appliquez une goutte sur une surface hydrophobe et utilisez-le immédiatement pour complètement remplir une microcuvette en une fois.
7. Essuyez le surplus de liquide de contrôle à l'extérieur de la microcuvette. Veillez à ne pas aspirer de liquide, contenu dans la microcuvette.
8. Faites une inspection visuelle :
   - Si la microcuvette n'est pas entièrement rempli, ne la remplissez pas une deuxième fois, mais jetez la et remplissez une nouvelle microcuvette.
   - Si la microcuvette contient des bulles d'air, jetez la et remplissez une nouvelle microcuvette.
9. Assurez-vous que l'l'appareil est en mode « Prêt ». Insérez la microcuvette remplie dans le support de microcuvette et appuyez. Le résultat s'affiche en une seconde.
   Attention : La microcuvette doit être insérée au maximum 40 secondes après avoir rempli la microcuvette.
10. Vérifiez que le résultat tombe dans la plage de référence :
    Hemotrol Low : 5.7 – 7.3 g/dl
    Hemotrol Normal : 10.7 - 13.1 g/dl
    Si le résultat ne tombe pas dans la plage de référence, nettoyez/désinfectez l'appareil HemoCue et répétez la contrôle de qualité. Si le résultat reste en dehors de la plage de référence, contactez l'investigateur principal ou, en cas d'absence, le superviseur de site ou le superviseur local.
11. Après avoir rempli une microcuvette, essuyez le liquide de contrôle du capuchon coloré avec un linge propre et rebouchez soigneusement le flacon.
12. Après usage, replacez le flacon dans les conditions de conservation indiquées.

Autotest
Savez qu'un autocontrôle électronique interne (« autotest »), qui vérifie l'unité optique, est effectué chaque fois que l'l'appareil est mis sous tension, lorsque le support de microcuvette est à nouveau inséré et toutes les heures lorsqu'il est utilisé.

4. Dépannage

Si vous obtenez un résultat inattendu, il y a plusieurs causes possibles, par exemple :

- Remplissage insuffisant ou non uniforme de la microcuvette.
- Bulles d'air dans la microcuvette.

Dans ce cas-ci, recommencez la procédure avec une nouvelle microcuvette.

En cas de problèmes techniques, les symboles sur l'écran vous guideront pour prendre des actions pour résoudre le problème. En cas d'autres problèmes, consultez le manuel d'utilisation.

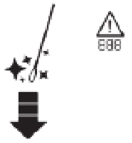	Nettoyez/Désinfectez l'appareil
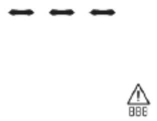 Au lieu d'un résultat	Recommencez la procédure avec une nouvelle microcuvette.
ECU	La microcuvette est soit vide ou mal insérée.<br><br>Recommencez la procédure avec une nouvelle microcuvette.
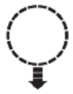	Il est possible que le support de microcuvette ne soit pas bien inséré, soit manquant ou cassé.<br><br>Assurez-vous que le support de microcuvette soit bien inséré.
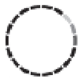	Si l'appareil est en permanence occupé.<br><br>Eteignez l'appareil et rallumez-le.
LLL ou HHH	Le résultat est inférieur à la plage de mesure (LLL) ou supérieur à la plage de mesure (HHH). – *Plage de mesure : 1.0 – 25.6 g/dl*<br><br>Recommencez la procédure avec une nouvelle microcuvette.<br><br>Si le deuxième test résulte de nouveau à LLL ou HHH, enregistrez le résultat ainsi et informez le clinicien traitants de l'enfant.
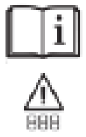 E00-E30	Retirez la microcuvette de son support.<br><br>Éteignez l'appareil HemoCue.<br><br>Nettoyez et désinfectez l'appareil.<br><br>Assurez-vous que l'appareil a atteint les conditions d'utilisation ambiantes avant démarrage. Evitez la lumière directe du soleil.<br><br>Rallumez l'appareil Hemocue.<br><br>Suivez toujours les informations et procédures décrites ici et dans le manuel d'utilisation.

5. Références

- Manuel d'utilisation de HemoCue 801

6. Histoire du document

Version 2.0 : Correction plage de référence Hemotrol
Ordre des échantillons et points d'attention pendant le prélèvement

Name and function	Date	Comments
Auteur original		
Bieke Tack<br>Version 2.0	25/11/2020<br>17/02/2021	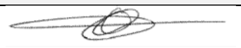
Révisé par		
Idzi Potters	17/12/2020	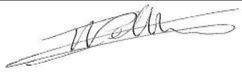
Approuvé par		
Jan Jacobs		
