# Supplementary material for: Field Experiences with Handheld Diagnostic Devices to Triage Children under Five Presenting with Severe Febrile Illness in a District Hospital in DR Congo
Source: Diagnostics (Basel). 2022 Mar 18;12(3):746. doi: 10.3390/diagnostics12030746 (PMC8947034; doi:10.3390/diagnostics12030746)
Supplement: Supplementary file 1 [file diagnostics-12-00746-s001.zip › Supplement Proofs/220317_BT_Field experiences_S1.pdf]

**Supplementary Figure S1.** Key elements in the lifecycle of handheld diagnostic devices at the health-facility level, based on the WHO Technical Series on Medical Devices to ensure improved access, quality and use of medical products and technology [7, 15-23, 26, 27, 57, 76].

### Needs assessment & selection:

**End-user:**

- Priority setting according to clinical needs, available financial & human resources, local standards & site-specific conditions (logistics, environment)
- Option appraisal:
  - Available products
  - Product evaluations, reported function & performance
  - Affordable total cost of ownership
  - Health technology assessments & target product profiles:
    - ✓ WHO priority medical devices [11]
    - ✓ WHO list essential in vitro diagnostics [12]
    - ✓ UNICEF supply catalogue [13]
- Product selection: quality & safety, effectiveness, appropriateness to local setting, usability, maintenance, affordability & availability (~ REASSURED criteria [14])

**Manufacturer/distributor:**

- Transparent communication of product specifications, availability & costs

### Procurement & shipment

**End-user:**

- Contract & order
- Receipt & check upon delivery

**Manufacturer/distributor:**

- Transparent & good governance
- Economically advantageous terms: right product, right quality, right price, right quantity, right place & time
- Satisfactory & well-defined terms for delivery, training, payment & warranty:
  - Local distributor/shipment via third party & customs
  - Storage & transport according to technical needs & regulations
  - Timely delivery & long shelf life

### Adoption, training & use

**End-user:**

- Installation according to local needs
- Adoption in local daily practice:
  - Integration in current practice & combination with other examinations
  - Standard Operating Procedures
- On-the-job & refresher training

**Manufacturer/distributor:**

- Clear instructions for use & appropriate labelling
- Regulatory approval, international standards, certification & calibration
- Satisfactory training & technical support

### Maintenance, repair & disposal

**End-user:**

- Equipment inventory & stock management
- Maintenance & storage: cleaning, disinfection, power supply, environmental conditions
- Regular quality control & calibration
- Disposal according to local guidelines
- Detect & document issues, provide feedback & follow manufacturer's instructions

**Manufacturer/distributor:**

- Satisfactory after-sales service:
  - Technical support
  - Procurement of consumables
  - Recalibration & maintenance
  - Warranty, estimated life span & availability of spare parts
  - Post-market surveillance & risk management:
    - ▪ Proactive collection, evaluation of and reaction to feedback from actual use, including product variability, environment / different user interaction, use(r) errors & unforeseen failure
    - ▪ Risk assessment, reporting & corrective actions if needed

**Total cost of ownership: purchase, delivery, installation & training, human resources, consumables, water & energy, maintenance & repair, disposal**
