# Supplementary material for: Field Experiences with Handheld Diagnostic Devices to Triage Children under Five Presenting with Severe Febrile Illness in a District Hospital in DR Congo
Source: Diagnostics (Basel). 2022 Mar 18;12(3):746. doi: 10.3390/diagnostics12030746 (PMC8947034; doi:10.3390/diagnostics12030746)
Supplement: Supplementary file 1 [file diagnostics-12-00746-s001.zip › Supplement Proofs/220317_BT_Field experiences_S2.pdf]

Supplement S2A. Semi-structured interview used to assess usability.

1. Adoption: training & integration
   - How was it to learn how to use the device?
   - How was it to integrate the device in the triage of children with severe febrile illness?
   - When was the last time you encountered problems with the integration of the device in the daily practice? Please explain what happened. Are there other problems that you encountered with the integration of the device? Which?
   - Overall, how do you evaluate the training process and integration of the device in daily practice? - Unsatisfactory, neutral, or satisfactory
2. Preparation:
   - How do you experience the preparation of the measurement?
   - How many steps do you need to perform during the preparation of the measurement?
   - How much time does the preparation of the measurement take?
   - When was the last time you encountered problems with the preparation of the measurement? Please explain what happened. Are there other problems that you encountered with the preparation of the measurement? Which?
   - Overall, how do you evaluate the preparation of the measurement? - Unsatisfactory, neutral, or satisfactory
3. Measurement:
   - How do you experience the measurement? How do you experience the specimen collection? How do you experience the analysis of the sample?
   - How many steps do you need to perform during the measurement?
   - How much time does the measurement take?
   - When was the last time you encountered problems with the measurement? Please explain what happened. Are there other problems that you encountered with the measurement? Which?
   - Overall, how do you evaluate the measurement? - Unsatisfactory, neutral, or satisfactory
4. Reading the result
   - What’s your opinion on the reading of result?
   - When was the last time you encountered problems with reading the result? Please explain what happened. Are there other problems that you encountered with the reading? Which?
   - Overall, how do you evaluate the measurement? - Unsatisfactory, neutral, or satisfactory
5. Maintenance
   - Have you ever cleaned the device? If yes, what’s your opinion on the cleaning of the device?
   - Have you ever changed the batteries of the device? How did this go?
   - Are there other maintenance activities that you have already undertaken? Or other maintenance activities which were required but you could not do by yourself? Which ones?
   - When was the last time you encountered problems with cleaning or maintenance? Please explain what happened. Are there other problems that you encountered with the cleaning or maintenance? Which ones?
   - Overall, how do you evaluate the maintenance? - Unsatisfactory, neutral, or satisfactory
6. Quality control & calibration

- Are there quality control actions that you have already undertaken with this device? Which ones? What's your opinion on these quality control actions?
- Have you ever felt the need to recalibrate a device? What did you do?
- When was the last time you encountered problems with quality control or calibration of a device? Please explain what happened. Are there other problems that you encountered with quality control or calibration? Which?
- Overall, how do you evaluate the quality control & calibration? - Unsatisfactory, neutral, or satisfactory

7. Hygiene & safety for HCW
    - How do you feel personally when using the device?
    - When was the last time you felt unsafe when using the device? Why? How often do you feel unsafe?
    - When was the last time you felt uncomfortable when using the device? Why? How often do you feel uncomfortable?
    - Overall, how do you evaluate your personal safety and comfort when using the device? - Unsatisfactory, neutral, or satisfactory
8. Safety & comfort for patient
    - When was the last time that the use of the device had a negative impact on a child's health? What happened? Did the same event occur in other children? Have you experienced other adverse effects related to the use of the device?
    - How does the child feel during the preparation and measurement with the device?
    - How does the caretaker feel during the preparation and measurement with the device?
    - How does the caretaker perceive and interprets what is displayed on the screen?
    - When was the last time a caretaker told you that they were worried about the safety of the device? What was he/she worried about? Did other caretakers express the same worries?
    - Overall, how do you evaluate the child's and his parent's safety and comfort of the device? - Unsatisfactory, neutral, or satisfactory
9. Size & weight , bed-side testing
    - What's your opinion on the size & weight of the device?
    - Would you recommend others to use this device for bedside testing? Why?
    - Overall, how do you evaluate the size and suitability for bed-side testing? - Unsatisfactory, neutral, or satisfactory
10. Overall usability:
    - What do you think of the device overall?
    - Do you have suggestions to improve the device?
    - Do you feel confident to use the device?
    - Do you have remaining questions or needs to use the device?
    - Overall, how do you evaluate the use of the device? - Unsatisfactory, neutral, or satisfactory
    - Irrespective of costs, would you buy the device again?

Supplement S2B. Label comprehension study in which seven end-users (5 nurses and 2 physicians) were asked to write down (open-ended response) the meaning of a selection of the pictorial symbols (labels, n = 20) depicted on the used diagnostic devices, their package or their IFU's. Numbers of nurses and physicians that correctly replied are reported below each label, meanings are reported in italics. The end-users were presented an empty black-and-white A4 print of the table of symbols below.

Question: Do you know what the following labels mean? They are printed on the diagnostic devices you have been using, on their package, or in their instructions for use.

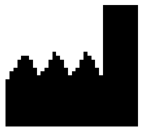<br>1 Nurse<br>*Manufacturer*	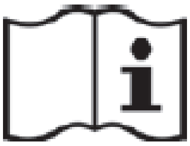<br>1 Physician<br>*Consult instructions for use*	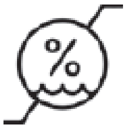<br>*Humidity limitation*	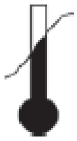<br>2 Nurses<br>1 Physician<br>*Temperature limitation*	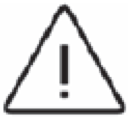<br>5 Nurses<br>2 Physicians<br>*Caution*
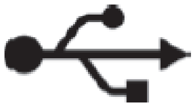<br>1 Physician<br>*USB port*	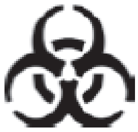<br>1 Nurse<br>*Biological risk*	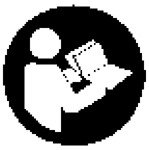<br>1 Physician<br>*Follow instructions for use*	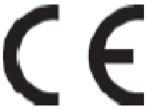<br>*CE mark*	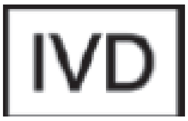<br>2 Nurses<br>*In vitro diagnostic medical device*
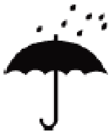<br>1 Nurse<br>1 Physician<br>*Keep dry*	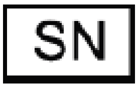<br>*Serial number*	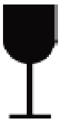<br>*Fragile, handle with care*	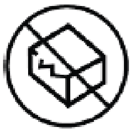<br>*Do not use if package is opened or damaged.*	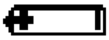<br>5 Nurses<br>2 Physicians<br>*Battery type*
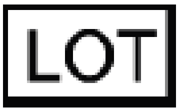<br>1 Physician<br>*Batch/lot code*	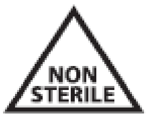<br>1 Nurse<br>2 Physicians<br>*Non-sterile*	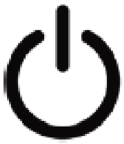<br>2 Nurses<br>2 Physicians<br>*Stand-by*	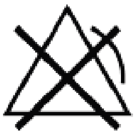<br>*No parameter alarms*	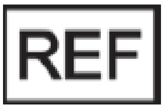<br>1 Nurse<br>2 Physicians<br>*Catalogue number*
